# Interventions to Mitigate the Effects of Housing Insecurity on Child and Adolescent Health: A Scoping Review

**DOI:** 10.3389/phrs.2025.1609177

**Published:** 2026-02-06

**Authors:** Artur Coy-Pérez, Juli Carrere, Anna Fernández, Carme Borrell, Gemma Serral, Esther Sánchez-Ledesma, Irene Macaya, Hugo Vásquez-Vera, Constanza Vásquez-Vera, Roshanak Mehdipanah, Katherine Pérez

**Affiliations:** 1 Agencia de Salut Publica de Barcelona, Barcelona, Spain; 2 Institut de Recerca Sant Pau, Barcelona, Spain; 3 Consorcio Centro de Investigacion Biomedica en Red, Madrid, Spain; 4 Pan American Health Organization, Washington, DC, United States; 5 Consorci de Salut i Social de Catalunya, Barcelona, Spain; 6 University of Michigan School of Public Health, Ann Arbor, MI, United States

**Keywords:** adolescents, children, health status, housing insecurity, interventions

## Abstract

**Objectives:**

This scoping review aimed to map and synthesize the available literature on interventions that mitigate the effects of housing insecurity on the health and wellbeing of children and adolescents (0–18 years), describing their characteristics, levels of action (structural, intermediate, or individual/group), and reported outcomes.

**Methods:**

In January 2025, we conducted a comprehensive search across four databases (PubMed, Scopus, Web of Science, and CINAHL) and 1 gray literature search engine (Carrot2), without time restrictions. 6,002 articles underwent three sequential screening phases. Results were described through a narrative synthesis of the evidence.

**Results:**

Twenty-six studies were included. Public housing, housing vouchers, and subsidies to private housing developers were the most common interventions, targeting structural and intermediate levels. Reported outcomes varied: physical health and healthcare use generally improved, while mental health and educational effects were mixed. Only two studies assessed multi-assistance programs.

**Conclusion:**

Affordability-focused interventions can improve health for children and adolescents, while multi-assistance approaches show promise. Broader welfare policies may also benefit this population. Future research should diversify geographically, use mixed methods, address age-specific outcomes, and examine more decommodifying housing strategies.

## Introduction

Housing insecurity is a form of residential exclusion that critically affects the health and wellbeing of children and adolescents (CAA). From a life course perspective, CAA’s health should be understood as the cumulative and dynamic result of social, emotional, and material exposures occurring from the earliest stages of life [1]. This perspective aligns with the World Health Organization’s (WHO) approach to health, which emphasizes the social determinants shaping the environments where individuals grow, form relationships, and develop [2]. In keeping with these frameworks, early-life adversities can trigger toxic stress processes that create a cumulative wear-and-tear effect on both physical and mental health [3, 4]. Evidence suggests that such stress undermines key aspects of CAA’s wellbeing during a critical period of development, affecting their ability to develop secure attachments, self-esteem, sense of agency, and engage in community life [5, 6]. The concept of embodiment reinforces this view by illustrating how social inequalities—including housing insecurity—become embedded in both the body and lived experience, contributing to the social gradient in health [7].

Likewise, housing has been recognized by the WHO as a key social determinant of health [8]. Building on a previous framework developed by Novoa et al. [9], Vásquez-Vera et al. [10] conceptualize the relationship between housing and health as mediated by a complex web of structural factors (such as macroeconomic policies, the housing market, and prevailing social values) that influence both the tangible (such as physical quality, affordability, and legal security) and intangible (including emotional safety and social connectedness) dimensions of housing. Neighborhoods may amplify or buffer these effects through their physical attributes and community features.

In this context, housing insecurity is defined as a multidimensional condition marked by unstable, unaffordable housing and risk of eviction that reflects both material and emotional dimensions [11]. It encompasses experiences of frequent and involuntary residential mobility, housing stress, overcrowding, and inadequate or non-tenured living situations (such as doubling-up or squatting) [11, 12]. Multiple studies have shown that housing insecurity negatively impacts various dimensions of CAA’s health and wellbeing through different mechanisms. Frequent residential moves disrupt routines, social networks, and school connections, causing stress, poorer socio-emotional development, more chronic health conditions, reduced healthcare coverage, and unmet health needs [13, 14]. Financial hardship related to housing costs has been linked to higher infant mortality, low birth weight, delayed medical care, and poorer academic performance, including reduced likelihood of completing higher education [13, 15]. Overcrowding and noise disrupt sleep, hinder study, and harm the mental health of both CAA and caregivers, increasing the risk of family conflict, neglect, and abuse [16–18]. Evictions, in turn, have been associated with preterm births, food insecurity, hospitalizations, poorer cognitive and emotional outcomes, and increased likelihood of child welfare system involvement [17, 19–21].

Accordingly, interventions that mitigate the effects of housing insecurity on the health and wellbeing of CAA can be understood as acting at structural, intermediate, or individual/group levels—corresponding, respectively, to broader policy and housing market dynamics, housing and neighborhood conditions, and the everyday living conditions that shape people’s direct experiences of housing, as conceptualized by Vásquez-Vera et al. [10]. This typology also reflects deeper distinctions in terms of their redistributive logic and their capacity to decommodify housing. Structural-level interventions, such as rent control, the expansion of public or non-market housing, or strengthened tenant protections, are strongly decommodifying in nature [22, 23], reducing reliance on the private market and altering structural conditions that produce housing insecurity. In contrast, intermediate-level interventions—such as housing subsidies, vouchers, or the coordination of housing and community services—primarily operate as redistributive mechanisms [24, 25], reallocating resources within existing market structures without challenging the commodified logic of housing provision. Finally, individual or group-level interventions—including psychosocial support, case management, or vocational training—focus on alleviating the personal and social consequences of housing insecurity without altering housing conditions, operating within a residual redistributive logic [26, 27].

Although several reviews have explored housing-related interventions affecting children and adolescents [28–34], most remain fragmented, focusing on specific housing provision programs such as vouchers or public housing. To date, no synthesis has systematically examined the full range of existing interventions, which encompasses not only different forms of housing provision but also community-based services, multi-assistance approaches, and interventions that work through supportive or psychosocial mechanisms rather than through changes to housing itself. Examining this broader spectrum is essential for understanding how the diverse strategies that currently address housing insecurity relate to one another and where important gaps remain.

Furthermore, no review has examined these interventions through a conceptual lens that distinguishes their level of action. Such a distinction is crucial, because structural, intermediate, and individual/group interventions target fundamentally different determinants of housing insecurity and operate through distinct decommodifying and redistributive logics. Distinguishing these levels helps clarify whether current efforts primarily mitigate the consequences of housing insecurity or meaningfully engage with the structural conditions that produce it. By applying this typology, our study provides a novel perspective that goes beyond describing isolated initiatives, offering a clearer understanding of how different approaches align with or challenge the structural drivers of housing insecurity, and assessing the transformative potential of interventions regarding health equity and housing decommodification.

The aim of this study was to map and synthesize the available literature on interventions that mitigate the effects of housing insecurity on the health and wellbeing of CAA, describing their characteristics, levels of action (structural, intermediate, or individual/group), and reported outcomes.

## Methods

### Study Design

We conducted a scoping review following the Joanna Briggs Institute framework [35] and the PRISMA extension for scoping reviews [36]. This literature review method is particularly useful for fields where research may be emerging or fragmented [37]. As no prior review has offered a comprehensive mapping of the diverse interventions that mitigate the health impacts of housing insecurity among CAA, and considering the limited number of studies addressing this topic holistically, this approach allowed us to explore the breadth, nature, and key characteristics of the available evidence. Moreover, the inclusion of gray literature was essential to capture otherwise overlooked evidence, providing a more balanced view of the available data, and reducing the impact of publication bias [38].

### Research Question

Applying the Population–Concept–Context (PCC) framework recommended for scoping reviews [35], we formulated the primary research question: “What is the available literature on interventions that mitigate the effects of housing insecurity on the health and wellbeing of children and adolescents?”

The research sub-questions were:

- What do these interventions consist of, and at which level (structural, intermediate, or individual/group) are they implemented?
- What are the reported effects of these interventions on the health and wellbeing of children and adolescents?

### Search Strategy, Screening and Selection

We performed the literature search in January 2025 across four major databases in the field of social sciences and health (PubMed, Scopus, Web of Science, and CINAHL) as well as a gray literature search engine (Carrot2), using English-language search terms and with no time restrictions. Similarly, in accordance with the JBI Manual for Evidence Synthesis [35], no language restrictions were applied; studies in any language were eligible for inclusion (and would have been translated if necessary). Carrot2 was included for its ability to cluster search results by topic, enhancing the retrieval of diverse and thematically organized gray literature [39]. The search strategy was developed based on keywords identified in the title and abstract of the articles obtained during an initial exploratory search. We structured the final search syntax around three core concepts, following the PCC framework: “children and adolescents” (Population), “housing insecurity” (Context), and “interventions” (Concept). Search strings were adapted to the specific syntax requirements of each database. Full details are provided in Supplementary Table 1.

To be eligible for inclusion, studies had to meet the following criteria: (1) involve children and adolescents (CAA) aged 0–18 years experiencing housing insecurity; and (2) describe and evaluate interventions with observable impacts on participants’ health and wellbeing. We included educational outcomes as part of our focus, as they are closely intertwined with the overall wellbeing of CAA and are recognized determinants of health and development [40]. We also operationalized housing insecurity to include situations of financial hardship due to housing costs, risk of eviction, squatting driven by financial need, and doubling-up (i.e., living with relatives or friends due to the lack or loss of one’s own housing) [12].

Studies were excluded if all participants were homeless, living in shelters, or residing in slum housing. According to the European Typology of Homelessness and Housing Exclusion (ETHOS) [41], homelessness includes both rooflessness (living in public spaces without shelter) and houselessness (residence in emergency or temporary accommodation). These situations fall below ETHOS’s minimum adequacy threshold, as individuals lack access to core elements of adequate housing—physical adequacy, legal security of tenure, and the ability to maintain privacy and social relations. In contrast, people experiencing housing insecurity may face instability or deficits in one or more of these domains (e.g., arrears, risk of eviction, limited privacy when doubling-up or squatting), yet they still occupy a dwelling that meets ETHOS’s basic adequacy criteria. By definition, rooflessness, houselessness, and slum housing—classified as “inadequate housing”—represent more severe forms of deprivation than housing insecurity. Including them would broaden the construct beyond our defined scope and reduce comparability with studies that distinguish housing insecurity from homelessness and extreme housing exclusion.

We also excluded studies where all participants were already stably housed through housing assistance (e.g., public housing, housing vouchers). Eligible sources included scientific publications (quantitative, qualitative, or mixed-methods empirical studies), government or private organization reports, doctoral theses, conference proceedings, letters to the editor, scientific communications, public policy documents, or clinical or social practice guidelines.

Reviewers independently screened a pilot sample of 20 records to calibrate their understanding of the inclusion criteria and ensure consistent application before beginning the formal screening. We conducted the document screening in three consecutive phases. The first one involved reviewing studies’ titles and abstracts, and the second one consisted of a full-text screening. In the third phase, we manually searched the reference lists of the selected documents—including those of literature reviews, which were not eligible sources—to identify additional studies meeting our inclusion criteria. To ensure internal validity, four independent pairs of researchers conducted the screening in phases one and two. Discrepancies were resolved through discussion, and if consensus could not be reached, a third researcher was consulted. The same procedure was applied in phase three, with two pairs of researchers conducting the review and triangulation. To assess the consistency of the screening process, we calculated inter-rater agreement for the title/abstract screening phase using Cohen’s kappa. Agreement between reviewer pairs was very high (κ = 0.94). The *Rayyan* software [42] was used throughout the document screening process to facilitate coordination among reviewers.

### Data Extraction and Quality Assessment

For each selected document, we extracted the following information: (a) study characteristics; (b) characteristics of the interventions; and (c) study quality. Study characteristics included the title, authors, year of publication, document type, study objectives, design, sample, target population, and instruments used to assess health and wellbeing impacts. Intervention characteristics included the country of implementation, description, duration, and health/wellbeing effects on CAA—which were based on significant changes. Additionally, we classified each intervention according to their level of action: (1) structural, for interventions that addressed structural determinants of housing insecurity by modifying supply, regulations, or tenant protections in ways that promote decommodification; (2) intermediate, for redistributive measures working within market systems; and (3) individual/group, for strategies addressing personal or social impacts without changing housing conditions. We also categorized each intervention into four broad types based on their nature: public housing, housing vouchers, subsidies to private developers of affordable housing, and multi-assistance programs.

Although formal quality appraisal is not typically required in scoping reviews [35], we conducted one to better understand the strength and reliability of the available evidence and to enhance transparency, given the inclusion of diverse source types—from social organization reports and conference proceedings to peer-reviewed articles—whose methodological rigor may vary. For this purpose, we used the Mixed Methods Appraisal Tool (MMAT) [43], a validated and widely used instrument [44–46] designed to assess a range of study designs, including qualitative studies, randomized controlled trials, non-randomized quantitative studies, quantitative descriptive studies, and mixed methods research. Each study was evaluated according to the criteria specific to its methodological category. The MMAT includes 25 items—five per study type—with response options: “Yes,” “No,” or “Can’t tell”. Following the tool’s authors recommendations [47], we calculated an overall quality score for each study based on the percentage of criteria met, and these scores were complemented with a descriptive assessment of the main methodological limitations.

## Results

**FIGURE 1:**
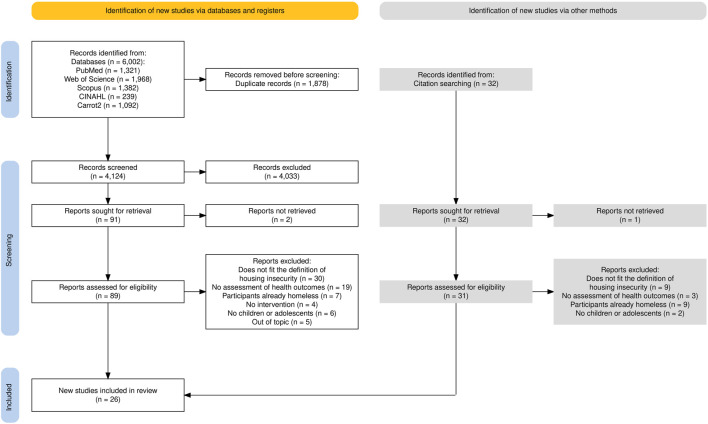
Flow diagram according to the Preferred Reporting Items for Systematic Reviews and Meta-Analysis extension for Scoping Reviews (Spain, 2025).

The database search yielded 6002 documents, with 1878 duplicates removed and 4033 excluded after title and abstract screening (Figure 1). After full-text review and reference list screening, 26 documents were included in the scoping review.

Table 1 summarizes the characteristics of the included documents. Most were published from 2010 onwards (73%) and were peer-reviewed articles (73.1%), while the remaining were gray literature, including reports, doctoral theses, and one letter to the editor. All but one study employed a quantitative design: eight were cross-sectional, fifteen longitudinal, one retrospective, and one a randomized controlled trial (RCT); the only mixed-methods study also incorporated an RCT design. All except one [48] included a comparison group. Half of them (53.9%) assessed two or three interventions.

**TABLE 1:** Description of included studies by year of publication, methods, document type, number of interventions studied, and study quality score, as well as description of interventions by their type, level of action, country, age of participating children and adolescents, study follow-up time, and health and wellbeing outcomes analyzed (Spain, 2025).

Characteristic	*N*	(%)
a) Characteristics of the studies	26	100.0
Year of publication		
Before 2000	2	8.0
2000–2009	5	19.0
2010–2020	13	50.0
After 2020	6	23.0
Document type		
Peer-reviewed article	19	73.1
Gray literature	7	26.9
- Report	4	15.4
- Doctoral thesis	2	7.7
- Letter to the editor	1	3.8
Methods		
Quantitative	25	96.2
- Cross-sectional with comparison group	8	30.8
- Longitudinal with comparison group	14	53.8
- Longitudinal without comparison group	1	3.8
- Randomized controlled trial	1	3.8
- Retrospective with comparison group	1	3.8
Mixed methods (randomized controlled trial + Semi-structured interviews)	1	3.8
Number of interventions included per study		
1	12	46.2
2	10	38.5
3	4	15.4
Study quality score		
100%	16	61.5
80%	6	23.1
60%	3	11.5
40%	1	3.9
b) Characteristics of interventions studied^a^		
Intervention type		
Public housing	16	61.5
Housing vouchers	16	61.5
Subsidies to private developers of affordable housing	9	34.6
Multi-assistance (legal, financial, medical, housing, and wraparound support)	2	7.7
Level of intervention		
Structural	16	61.5
Intermediate	22	84.6
Individual/group	2	7.7
Country of intervention		
USA	25	96.2
Ecuador	1	3.8
Age of participating children and adolescents		
0–2 years	14	53.8
3–5 years	11	42.3
6–12 years	13	50.0
13–17 years	13	50.0
Unspecified	4	15.4
Study follow-up time		
No follow-up	8	30.8
2 years or less	7	26.9
3–9 years	5	19.2
10 years or more	6	23.1
c) Health and wellbeing outcomes^b^		
General health status	3	11.5
Physical health		
Health conditions (e.g., asthma, skin allergies, chronic illnesses)	7	26.9
Nutrition/Growth	5	19.2
Mental health		
Psychological distress (i.e., anxiety, depression, internalizing symptoms)	4	15.4
Behavior (i.e., externalizing symptoms, substance use)	6	23.1
Maltreatment outcomes (i.e., reports of negligence and physical or sexual abuse)	2	7.7
Healthcare access and utilization		
Preventive care (e.g., check-ups, dental visits)	1	3.8
Urgency care	4	15.4
Hospitalizations	6	23.1
Educational outcomes	​	​
School attendance	5	19.2
Short-term academic metrics (i.e., grades and progression)	7	26.9
Long-term educational attainment	4	15.4

^a^ Several studies fall into more than one category; percentages indicate the proportion of the total 26 studies that included each intervention characteristic.

^b^ Several studies considered more than one type of health-related outcomes; percentages indicate the proportion of the total 26 studies in which the health outcome was analyzed.

As for the study quality scores, sixteen scored the maximum [49–64], while six scored 80% [48, 65–69], three scored 60% [70–72], and one scored 40% [73]. Across the ten studies scoring below 100%, the most frequent limitations reflected risks of bias related to confounding, completeness of data, and intervention delivery. Studies using randomized quantitative designs [65, 66] lacked blinded outcome assessment, which may have influenced how outcomes were measured and interpreted, even though other aspects of the design were robust. Non-randomized observational studies [48, 67–73] more often failed to account adequately for potential confounders [67, 69, 70, 73], had incomplete or differentially missing outcome data [68, 72, 73], or showed problems with how the intervention was delivered or targeted [71, 73], which limits internal validity and generalizability. Some differences were also apparent by document type: reports [65, 67, 70] and a letter to the editor [73] tended to provide less detailed methodological reporting, while peer-reviewed articles [48, 66, 68, 71, 72] and a doctoral thesis [69] described methods more fully but still exhibited constraints related to adherence and implementation of the intervention [71, 73], sample representativeness and attrition [71, 72], and the use of aggregated ecological data [48], which limits causal inference.

Most studies evaluated public housing and housing vouchers interventions (n = 16 each), while approximately one-third examined subsidies to private developers of affordable housing (n = 9), and two assessed multi-assistance programs. Interventions’ level of action was primarily intermediate (n = 22), followed by structural (n = 16), and individual/group (n = 2). All interventions were US-based, except one in Ecuador. Fourteen studies included CAA aged 0–2, eleven aged 3-5, thirteen aged 6–12, and thirteen aged 13–17; four did not report age. Follow-up lasted 2 years or less in seven studies, 3–9 years in five, 10 years or more in six, and there was no follow-up in eight studies.

Outcomes assessed included general and physical health, mental health (psychological distress and behavior), and maltreatment. Studies also examined healthcare use—such as urgency care, hospitalizations, and preventive care. Educational outcomes included short-term academic metrics, school attendance, and long-term educational attainment.

Supplementary Table 2 presents the studies’ aims, methodologies, instruments, and quality scores, along with descriptions of the interventions studied and main results. Table 2 summarizes the observed effects of the interventions on CAA’s health and wellbeing outcomes.

**TABLE 2:** Number of studies reporting beneficial, neutral or detrimental effects of interventions on health and wellbeing outcomes, by intervention type (Spain, 2025).

Outcome	Intervention type											
	Public housing			Housing vouchers			Subsidies to private developers of affordable housing			Multi-assistance (legal, financial, medical, housing, and wraparound support)		
	Beneficial	Neutral	Detrimental	Beneficial	Neutral	Detrimental	Beneficial	Neutral	Detrimental	Beneficial	Neutral	Detrimental
**General health status**	1 (March et al. [70])	1 (Meyers et al. [60])	​	1 (March et al. [70])	1 (Meyers et al. [60])	​	​	​	​	1 (Bovell-Amon et al. [66])	​	​
**Physical health**												
Health conditions (e.g., asthma, skin allergies, chronic illnesses)	3 (Fenelon et al. [53]; Meyers et al. [73]; Turcotte et al. [72])	2 (Boudreaux et al. [49]; Fenelon [52])	​	1 (Fenelon et al. [53])	1 (Boudreaux et al. [49])	​	1 (Fenelon et al. [53])	1 (Boudreaux et al. [49])	1 (Gensheimer et al. [55])	1 (Bovell-Amon et al. [66])	​	​
Nutrition/Growth	3 (March et al. [70]; Meyers et al. [59]; Meyers et al. [60])	1 (Meyers et al. [73])	​	3 (March et al. [70]; Meyers et al. [59]; Meyers et al. [60])	​	​	​	​	​	​	1 (Bovell-Amon et al. [66])	​

**Mental health**												
Psychological distress (i.e., anxiety, depression, internalizing symptoms)	3 (Coley et al.^a^ [50]; Fenelon et al. [54]; Turcotte et al. [72])	2 (Coley et al.^a^ [50]; Fenelon et al.^a^ [54])	1 (Musa et al. [61])	2 (Coley et al.^a^ [50], Fenelon et al. [54])	2 (Coley et al.^a^ [50]; Fenelon et al.^a^ [54])	1 (Musa et al. [61])	1 (Fenelon et al. [54])	1 (Fenelon et al.^a^ [54])	​	​	​	​
Behavior (i.e., externalizing symptoms, substance use)	2 (Fenelon et al. [54]; Newman and Holupka,^a^ [68])	5 (Coley et al. [50]; Fenelon et al.^a^ [54]; Leech [57]; Musa et al. [61]; Newman and Holupka [68])	1 (Newman and Holupka,^a^ [68])	3 (Fenelon et al. [54]; Leech [57]; Newman and Holupka, 2016^a^ [68])	5 (Abt Associates et al. [65]; Coley et al. [50]; Musa et al. [61]; Fenelon et al.^a^ [54]; Newman & Holupka [68])	1 (Newman and Holupka,^a^ [68])	2 (Fenelon et al. [54]; Newman and Holupka,^a^ [68])	2 (Fenelon et al.^a^ [54]; Newman and Holupka [68])	1 (Newman and Holupka,^a^ [68])	​	​	​

**Maltreatment outcomes (i.e., reports of negligence and physical or sexual abuse)**	​	​	​	1 (Pergamit et al.^a^ [71])	​	1 (Pergamit et al.^a^ [71])	​	1 (Ports et al. [48])	​	​	​	​

**Healthcare access and utilization**												
Preventive care (e.g., check-ups, dental visits)	​	​	​	​	​	​	1 (Gensheimer et al. [55])	​	​	​	​	​
Urgency care	2 (Boudreaux et al. [49]; Fenelon et al. [53])	​	​	2 (Boudreaux et al. [49]; Fenelon et al. [53])	1 (Jacob et al. [56])	​	2 (Boudreaux et al. [49]; Fenelon et al. [53])	​	​	​	1 (Bovell-Amon et al. [66])	​
Hospitalizations	3 (Fenelon et al. [53]; Sandel et al. [63]; Turcotte et al. [72])	1 (Meyers et al. [60])	​	2 (Fenelon et al. [53]; Sandel et al. [63])	2 (Jacob et al. [56]; Meyers et al. [60])	​	2 (Fenelon et al. [53]; Sandel et al. [63])	​	​	​	1 (Bovell-Amon et al. [66])	​

**Educational outcomes**												
School attendance	1 (Fenelon et al. [53])	​	​	2 (Abt Associates et al. [65]; Fenelon et al. [53])	1 (Fenelon et al.^a^ [53])	​	2 (Fenelon et al. [53]; Liaw [58])	​	1 (Gensheimer et al. [55])	1 (Herzberg et al. [67])	​	​
Short-term academic metrics (i.e., grades and progression)	3 (Coley et al.^a^ [50]; Currie and Yelowitz [51]; Newman and Holupka^a^ [68])	3 (Coley et al.^a^ [50]; Currie and Yelowitz,^a^ [51]; Newman and Holupka [68])	1 (Newman and Holupka,^a^ [68])	3 (Coley et al.^a^ [50]; Newman and Holupka [68]; Schwartz et al. [64])	4 (Coley et al.^a^ [50]; Jacob et al. [56]; Newman and Holupka [68]; Schwartz et al., 2019* [64])	2 (Abt Associates et al. [65]; Newman and Holupka,^a^ [68])	2 (Liaw [58]; Newman and Holupka,^a^ [68])	1 (Newman & Holupka [68])	2 (Liaw [58]; Newman and Holupka^a^ [68])	​	​	​
Long-term educational attainment	​	1 (Newman and Harkness [62])	​	1 (Rosero [69])	3 (Abt Associates et al. [65]; Jacob et al. [56]; Rosero [69])	​	​	1 (Newman and Harkness [62])	​	​	​	​

^a^ Health and wellbeing outcomes marked with an asterisk are not generalizable and apply only to a specific subgroup within the study population.

### Public Housing

At the structural level of action, we found public housing interventions (n = 16)—government-owned, fully subsidized units allocated based on income criteria, with rent typically limited to 30% of household earnings [51]. Ten studies examining public housing outcomes for CAA found consistent physical health benefits. Some reported reduced risks of ear infections [53] and iron deficiency [73], while one showed better asthma-related health scores compared to non-assisted peers [72]. Regarding nutrition, two studies documented improved growth indicators (weight-for-age/height) [59, 60], others found lower underweight risk [59, 70], and one reported reduced food insecurity [70]. However, two studies reported no significant effects on physical health outcomes [52, 68], one found no change in asthma attack incidence [49], and another showed no improvement in growth metrics [73].

Mental health findings presented a more complex picture. Two studies found fewer internalizing symptoms [54] and emotional difficulties [54, 72] among CAA in public housing, and one longitudinal study showed slower progression of anxiety/depression symptoms with age [50]. However, one contrasting study reported increased psychological distress [61]. Three studies concurred on null effects for externalizing behaviors [50, 61, 68], while one found no substance use differences [57].

Regarding healthcare use, three studies found reduced hospitalizations [53, 63, 72] and two reported fewer asthma-related urgency care visits [49, 53], though another found no change in service utilization [60]. Educational outcomes were similarly mixed. Two studies showed reduced absenteeism [53] and improved adolescent math performance [50], while one study found lower grade repetition rates [51]. However, quantile analysis in one study revealed cognitive benefits limited to high-performing CAA [68], and another found no impact on high school graduation rates [62]. Additionally, one study noted higher likelihood of being classified as having good general health status [70].

### Housing Vouchers

Housing voucher programs (n = 16) offer direct rental subsidies to low-income families, allowing them to access housing in the private market while contributing only a capped portion of their income [64, 69]. Regarding mental health outcomes, multiple studies found children in voucher programs exhibited fewer symptoms of psychological distress compared to those without subsidies [50, 54], along with slower emergence of such symptoms over time [50] and reduced substance use [57]. However, five studies reported no significant effects on behavioral or externalizing problems [50, 54, 61, 65, 68], and one study found detrimental mental health impacts [61].

Studies showed consistent healthcare benefits, with three reporting fewer asthma-related visits [49, 53] and reduced hospitalizations [53, 63], though two studies found no significant differences in utilization [56, 60]. Benefits were also reported for physical health outcomes: three studies documented nutritional improvements, including reduced underweight prevalence and food insecurity [59, 60, 70], along with better overall health status [70] and lower ear infection risk [53]. Other studies, however, found no significant physical health associations [49, 60, 68].

Educational impacts varied. Voucher recipients showed fewer school absences [53, 65], modest grade improvements in English [64] and math [50, 64], and higher adolescent school enrollment rates [69]. Yet other studies reported null effects on grades [56] and educational attainment [56, 65], with mixed cognitive outcomes by performance level [68] and increased grade repetition risk [65].

The single study examining families at risk of foster care involvement or unable to reunify due to housing insecurity revealed complex patterns: while child maltreatment reports decreased among families preventing separation, reunified families showed variations with both increases and decreases [71].

### Subsidies to Private Developers of Affordable Housing

At the intermediate level, some interventions provided financial incentives—such as subsidies, tax credits, or low-interest loans—to private developers to deliver and market affordable housing units (n = 9) [55, 62]. Research on CAA in subsidized units revealed mixed educational impacts. Findings included attendance and grade improvements when school continuity was maintained, although school changes were associated with reduced absenteeism but lower Math performance [58]. Other studies found higher chronic absenteeism rates [55] or no significant effects on cognitive yield or long-term educational attainment [62, 68].

Health outcomes showed some benefits, including lower rates of ear infections and reductions in asthma-related urgency care visits [49, 53], in hospitalizations [53, 63], and in school absences [53, 58]. Improved healthcare utilization was noted through increased pediatric check-ups and dental visits [55]. However, no improvements were observed in psychological wellbeing [54], behavior [54, 68], or maltreatment [48]. In addition, while one study reported higher asthma risk [55], others found no impact [68].

### Multi-Assistance

At both the intermediate and individual/group levels, we identified multi-assistance interventions (n = 2), which combine community coordination—linking housing, healthcare, education, and legal services—with personalized, wraparound support tailored to each family’s needs [66, 67]. One study found CAA taking part in a multi-assistance intervention showed better overall health status and typical developmental progress compared to non-assisted peers, alongside reduced emergency visits and hospitalizations in both groups, though nutritional outcomes remained unchanged [66]. A separate study reported that rental assistance alone improved school attendance, while supplemental services (educational support, healthcare access, counseling, and community programs) provided no additional benefits [67].

## Discussion

This review is the first to map the available literature on interventions that mitigate the effects of housing insecurity on the health and wellbeing of CAA. Structural-level interventions, specifically public housing, showed consistent positive effects on physical health, healthcare utilization, and nutritional outcomes. Intermediate-level interventions, including housing vouchers and subsidies to private developers, were most frequently evaluated and yielded mixed but sometimes positive effects—particularly on healthcare use and educational metrics—though mental health and long-term educational attainment outcomes were inconsistent. No interventions operated exclusively at the individual or group level. However, multi-assistance programs combining psychosocial or family support with intermediate measures (e.g., housing subsidies) showed promising effects on health and school attendance. Their value appears to lie in enhancing the impact of intermediate interventions by addressing families’ specific social needs.

As for the observed benefits of the interventions, it is important to note that all of them, regardless of their level of action, share a focus on housing assistance and affordability. These interventions contribute to financial and residential stability by redistributing housing costs away from low-income families, enabling greater investment in children’s health, nutrition, and education [74, 75]. Crucially, structural interventions like public housing also carry a degree of decommodification, as they expand the non-market supply of housing and reduce exposure to market volatility—thereby providing a more stable and secure foundation for CAA wellbeing. These interventions—whether redistributive within market frameworks or more structurally transformative—can help buffer families from the residential mobility linked to housing insecurity, which has been proven to be a major source of stress linked to poor health outcomes [13, 14].

Regarding the few harms attributed to the interventions, some authors point to uncontrolled factors, such as neighborhood characteristics (e.g., safety or collective efficacy) [61], as well as the disruptive effects on social networks caused by interventions that involve family relocation, like housing vouchers [55]. We must also consider that, even with comparable control groups and adjustments for socioeconomic factors, comparing families receiving housing assistance with those who do not—as seen in studies with negative outcomes [55, 61, 68]—may still introduce selection bias. Families entering assistance programs are more likely to have lived in substandard housing, and their underlying motivations for seeking aid are often unobserved [51]. These pre-existing disadvantages may also make their children more prone to negative outcomes independent of the intervention. Precisely, one study found that assisted housing had divergent effects on behavioral and cognitive outcomes depending on children’s baseline performance [68], suggesting that those already facing greater adversity may benefit less or even be negatively affected by the intervention. This underscores the importance of early intervention in housing insecurity, as evidence suggests that while impacts can be mitigated, the cumulative effect of long-term adversity can still limit intervention effectiveness [28].

In contrast, only positive effects were observed in the two articles studying multi-assistance interventions. Despite the limited scope of individual/group-level interventions, we hypothesize that combining holistic support services—such as healthcare, education, and case management—with intermediate-level housing affordability measures can enhance CAA wellbeing, although only one study strongly supports this. The near absence of qualitative studies further limits understanding of lived experiences and mechanisms through which interventions impact CAA health. Qualitative research is essential to capture affected populations’ voices and understand the pathways linking interventions to wellbeing [76], including why similar interventions may yield different effects across contexts and population subgroups.

Similarly, many interventions potentially beneficial to CAA’s health and wellbeing lack impact evaluation on health outcomes and therefore are not included in this review. Nonetheless, policies addressing housing affordability, financial stability, and social inclusion are likely to improve health outcomes. This applies to structural- and intermediate-level measures such as mortgage regulation to prevent borrower abuse, household debt refinancing [9], foreclosure prevention counseling [77], or “inclusionary zoning” policies requiring new developments to include affordable units [78]. Anti-speculation and decommodifying policies like rent control [79], rent stabilization measures [77], and second-home purchasing restrictions [80] have also been associated with reduced housing costs and improved access for low-income households. Moreover, broader welfare state measures—universal healthcare, labor integration programs, unemployment benefits, and anti-exclusion policies—may also help mitigate the health impacts of housing insecurity by reducing financial hardship, protecting against mental distress, and maintaining access to essential services [81, 82].

This review has also excluded widely studied programs like Moving to Opportunity, as it targets families already stabilized through housing assistance. This intervention relocates low-income families to low-poverty areas using vouchers [83], but its mixed effects [84] and the criticism it has received for neglecting structural drivers of housing insecurity and disrupting vital social networks [85, 86] are significant.

Aside from this, our findings reveal a marked increase in the number of published studies from 2009 onward, possibly spurred by heightened attention to the topic following widespread housing insecurity during the global financial crisis. We also observe an overwhelming predominance of US-based studies, consistent with prior evidence [87]. This concentration mirrors the broader characteristics of the country’s liberal welfare regime [88], where housing is primarily treated as a market commodity and public interventions are residual and targeted [89]. These frameworks tend to favor redistribution without challenging the commodified nature of housing [24], constraining their capacity to address structural determinants of CAA health. More substantial effects might arise in contexts with stronger decommodification and universal welfare protections. Notably, no housing interventions have been evaluated for health impacts in such contexts.

Due to the USA’s dominance, this review has also unintentionally given greater weight to interventions like housing vouchers and subsidies to private developers, widely implemented and studied there [74]. These approaches reflect a broader neoliberal shift in the USA housing policy, where the state has reduced direct provision and turned to market-based solutions [90]. By channeling public funds into private housing markets, these programs frame housing as a commodity rather than a social right [91, 92]. While redistributive in nature, they preserve the commodified structure of provision—raising doubts about their ability to address the root causes of housing-related health inequalities.

Lastly, the current body of evidence is also characterized by a lack of age-disaggregated data. Most interventions targeted wide age ranges without differentiating between children and adolescents, making it impossible to assess potential differences in how these groups experience and benefit from them. Given the distinct developmental and social needs of these age groups, this gap significantly constrains the ability to capture age-specific outcomes—an important consideration for future evaluations.

### Strengths and Limitations

This scoping review allowed for an exploration of the complex and multidimensional issue of housing insecurity and its effects on CAA’s health, covering a wide range of intervention types and evidence sources. This breadth not only builds a more comprehensive understanding of interventions studied to date but also addresses a notable gap in the literature, as—to our knowledge—no other review has examined this topic with comparable scope.

A key strength of this review is the inclusion of gray literature, which helps mitigate publication bias by capturing evidence unpublished in peer-reviewed journals [38]. While our search was English-only, most non-English publications include English abstracts, and tools like Carrot2 use AI capable of cross-lingual retrieval. Nevertheless, the reliance on English-language interfaces and indexing likely favored the retrieval of studies published in English and may have contributed to the under-representation of evaluations conducted and reported in other languages and settings. This language focus limits the geographical breadth of the available evidence and constrains the generalizability of the findings to other welfare and housing regimes. Future reviews could improve comprehensiveness and geographical diversity by incorporating targeted multilingual searches.

Besides, our operational definition of housing insecurity—focused on affordability problems, tenure instability, doubling-up, and eviction risk—necessarily excluded interventions aimed at improving CAA’s health and wellbeing through other dimensions of housing. As a result, programs addressing issues such as the physical quality of housing may have been overlooked, even though they often serve populations experiencing forms of housing insecurity that are not explicitly labeled as such. Addressing all these gaps would contribute to a fuller and more nuanced picture of the ways in which interventions targeting housing insecurity influence health and wellbeing among CAA.

### Conclusion

This review highlights the potential of affordability-focused interventions to reduce the adverse effects of housing insecurity on CAA’s wellbeing, primarily through enhanced financial and residential stability. While many programs operate within market-based frameworks—such as housing vouchers and subsidies to private developers—public housing stands out for its more decommodifying role. Redistributive approaches offer short-term benefits, particularly in physical health, mental health, and education. However, market-based interventions may limit structural impact and sometimes disrupt social networks or overlook deeper inequalities. By contrast, multi-assistance interventions—though fewer—show promise, likely due to integrating housing with broader social supports. At the same time, other structural policies—such as rent control, debt regulation, or welfare supports—may also benefit CAA, even if their impacts remain unevaluated. Despite growing evidence, significant gaps persist: few studies come from outside the USA, and research on universalist welfare contexts is scarce. The lack of qualitative and participatory approaches limits understanding of CAA’s lived experiences. Advancing the field requires greater geographical diversity, more mixed methods, and closer attention to age-specific effects of housing insecurity. Future work should also explore decommodifying strategies and their potential to create lasting improvements in CAA’s wellbeing through comprehensive supports.

## References

[1] Kelly-Irving M . Allostatic Load: How Stress in Childhood Affects Life-Course Health Outcomes. In: Young People’s Future Health Inquiry (Future Health Inquiry). Report No.: 3 (2019). Available online at: https://www.health.org.uk/publications/allostatic-load (Accessed November 30, 2024).

[2] World Health Organization. Constitution of the World Health Organization. Geneva (1948). Available online at: https://www.who.int/about/governance/constitution (Accessed November 28, 2024).

[3] Shonkoff JP Garner AS Siegel BS Dobbins MI Earls MF McGuinn L The Lifelong Effects of Early Childhood Adversity and Toxic Stress. Pediatr (2012) 129(1):e232–46. 10.1542/peds.2011-2663 22201156

[4] Sterling P Eyer J . Allostasis: A New Paradigm to Explain Arousal Pathology. In: Fisher S Reason J , editors. Handbook of Life Stress, Cognition and Health. New York: John Wiley & Sons (1988). p. 629–49.

[5] Fattore T Mason J Watson E Mason J Watson E . When Children Are Asked About Their Well-Being: Towards a Framework for Guiding Policy. Child Indic Res (2008) 2(1):57–77. 10.1007/s12187-008-9025-3

[6] Bethell CD Newacheck P Hawes E Halfon N . Adverse Childhood Experiences: Assessing the Impact on Health and School Engagement and the Mitigating Role of Resilience. Health Aff (2014) 33(12):2106–15. 10.1377/hlthaff.2014.0914 25489028

[7] Krieger N . Embodiment: A Conceptual Glossary for Epidemiology. J Epidemiol Community Health (1978) (2005) 59(5):350–5. 10.1136/jech.2004.024562 15831681 PMC1733093

[8] Solar O Irwin A . A Conceptual Framework for Action on the Social Determinants of Health. In: Social Determinants of Health Discussion Paper 2 (Policy and Practice). Geneva: World Health Organization (2010). Available online at: https://www.who.int/publications/i/item/9789241500852 (Accessed November 29, 2024).

[9] Novoa AM Bosch J Díaz F Malmusi D Darnell M Trilla C . El Impacto de la Crisis en la Relación Entre Vivienda y Salud. Políticas de Buenas Prácticas Para Reducir las Desigualdades en Salud Asociadas Con Las Condiciones de Vivienda. Gac Sanit (2014) 28(S1):44–50. 10.1016/j.gaceta.2014.02.018 24863993

[10] Vásquez-Vera C Fernández A Borrell C . Gender-Based Inequalities in the Effects of Housing on Health: A Critical Review. SSM Popul Health (2022) 17:101068. 10.1016/j.ssmph.2022.101068 35360438 PMC8961216

[11] Hulse K Saugeres L . Housing Insecurity and Precarious Living: An Australian Exploration. Melbourne: Australian Housing and Urban Research Institute (2008). Available online at: https://www.ahuri.edu.au/research/final-reports/124 (Accessed November 17, 2024).

[12] Amore K Baker M Howden-Chapman P . The ETHOS Definition and Classification of Homelessness: An Analysis. Eur J Homelessness (2011) 5(2):19–37. Available online at: https://www.feantsa.org/research/journal/volume-5-issue-2 (Accessed November 24, 2024).

[13] Bess KD Miller AL Mehdipanah R . The Effects of Housing Insecurity on Children’s Health: A Scoping Review. Health Promot Int (2022) 38(3):1–11. 10.1093/heapro/daac006 35134939

[14] Baker E Pham NTA Daniel L Bentley R . How Does Household Residential Instability Influence Child Health Outcomes? A Quantile Analysis. Int J Environ Res Public Health (2019) 16(21):4189. 10.3390/ijerph16214189 31671903 PMC6862481

[15] Broton KM . Poverty in American Higher Education: The Relationship Between Housing Insecurity and Academic Attainment. JPSS (2021) 1(2):18–45. 10.33009/fsop_jpss129147

[16] Solari CD Mare RD . Housing Crowding Effects on Children’s Wellbeing. Soc Sci Res (2012) 41(2):464–76. 10.1016/j.ssresearch.2011.09.012 23017764 PMC3805127

[17] Clair A . Housing: An Under-Explored Influence on Children’s Well-Being and Becoming. Child Indic Res (2018) 12(2):609–26. 10.1007/s12187-018-9550-7

[18] Doan SN Evans GW . Chaos and Instability from Birth to Age Three. Future Child (2020) 30(2):93–114. 10.1353/foc.2020.a807753

[19] Schwartz GL Leifheit KM Chen JT Arcaya MC Berkman LF . Childhood Eviction and Cognitive Development: Developmental Timing-Specific Associations in an Urban Birth Cohort. Soc Sci Med (2022) 292:114544. 10.1016/j.socscimed.2021.114544 34774367

[20] Cutts DB de Cuba SE Bovell-Ammon A Wellington C Coleman SM Frank DA Eviction and Household Health and Hardships in Families with Very Young Children. Pediatr (2022) 150(4):e2022056692. 10.1542/peds.2022-056692 36120757

[21] Goplerud DK Leifheit KM Pollack CE . The Health Impact of Evictions. Pediatr (2021) 148(5):e2021052892. 10.1542/peds.2021-052892 34675132

[22] Marcinkiewicz E . Housing Decommodification vs. Housing Outcomes: A Comparative Study of the European Countries. Eur J Soc Sci (2023) 1–21. 10.1080/13511610.2023.2182221

[23] Kumnig S Litschauer K . Decommodified Housing Under Pressure: Contested Policy Instruments and Provisioning Practices in Vienna. Int J Hous Policy (2025) 1–23. 10.1080/19491247.2025.2458389

[24] Torgersen U . Housing: The Wobbly Pillar Under the Welfare State. Scand Hous Plann Res (1987) 4(Suppl. 1):116–26. 10.1080/02815737.1987.10801428

[25] Brenner N Theodore N . Cities and the Geographies of “Actually Existing Neoliberalism.”. Antipode (2002) 34(3):349–79. 10.1111/1467-8330.00246

[26] Parsell C Watts B . Charity and Justice: A Reflection on New Forms of Homelessness Provision in Australia. Eur J Homelessness (2017) 11(2):65–76. Available online at: https://www.feantsa.org/research/journal/volume-11-issue-2 (Accessed May 4, 2025).

[27] Shinn M. Homelessness , Poverty and Social Exclusion in the United States and Europe. Eur J Homelessness (2010) 4(1):19–44. Available online at: https://www.feantsa.org/research/journal/volume-4-2010 (Accessed May 4, 2025).

[28] Dunn JR . Housing and Healthy Child Development: Known and Potential Impacts of Interventions. Annu Rev Public Health (2020) 41:381–96. 10.1146/annurev-publhealth-040119-094050 31874071

[29] Slopen N Fenelon A Newman S Boudreaux M . Housing Assistance and Child Health: A Systematic Review. Pediatr (2018) 141(6):e20172742. 10.1542/peds.2017-2742 29765008 PMC6662196

[30] Aratani Y Lazzeroni S Brooks-Gunn J Hernández D . Housing Subsidies and Early Childhood Development: A Comprehensive Review of Policies and Demonstration Projects. Hous Policy Debate (2019) 29(2):319–42. 10.1080/10511482.2018.1515099

[31] Finnie RKC Peng Y Hahn RA Schwartz A Emmons K Montgomery AE Tenant-Based Housing Voucher Programs: A Community Guide Systematic Review. J Public Health Manag Pract (2022) 28(6):E795–803. 10.1097/phh.0000000000001588 36194822 PMC9555591

[32] Dockery AM Kendall G Li J Mahendran A Ong R Strazdins L . Housing and Children’s Development and Wellbeing: A Scoping Study. AHURI Final Rep No. 149 (2010). Available online at: https://www.ahuri.edu.au/research/final-reports/149 (Accessed May 6, 2025).

[33] Holme JJ . Growing up as Rents Rise: How Housing Affordability Impacts Children. Rev Educ Res (2022) 92(6):953–95. 10.3102/00346543221079416

[34] Newman SJ Holupka CS . Housing Affordability and Child Well-Being. Hous Policy Debate (2015) 25(1):116–51. 10.1080/10511482.2014.899261

[35] Peters MD Godfrey C McInerney P Munn Z Tricco AC Khalil H . Scoping Reviews. In: Aromataris E Lockwood C Porritt K Pilla B Jordan Z , editors. JBI Manual for Evidence Synthesis. Adelaide: JBI (2020). 10.46658/JBIMES-24-09

[36] Tricco AC Lillie E Zarin W O’Brien KK Colquhoun H Levac D PRISMA Extension for Scoping Reviews (PRISMA-ScR): Checklist and Explanation. Ann Intern Med (2018) 169(7):467–73. 10.7326/m18-0850 30178033

[37] Peters MD Marnie C Tricco AC Pollock D Munn Z Alexander L Updated Methodological Guidance for the Conduct of Scoping Reviews. JBI Evid Synth (2020) 18(10):2119–26. 10.11124/jbies-20-00167 33038124

[38] Paez A . Gray Literature: An Important Resource in Systematic Reviews. J Evid Based Med (2017) 10(3):233–40. 10.1111/jebm.12266 28857505

[39] Osiński S Weiss D . Carrot2: Design of a Flexible and Efficient Web Information Retrieval Framework. In: Szczepaniak PS Kacprzyk J Niewiadomski A , editors. Advances in Web Intelligence. Berlin, Heidelberg: Springer Berlin Heidelberg (2005). p. 439–44. 10.1007/11495772_68

[40] Raghupathi V Raghupathi W . The Influence of Education on Health: An Empirical Assessment of OECD Countries for the Period 1995-2015. Arch Public Health (2020) 78(20):1–18. 10.1186/s13690-020-00402-5 32280462 PMC7133023

[41] FEANTSA. How to Measure and Monitor Homelessness at EU Level (2010). Available online at: https://www.feantsa.org/en/feantsa-position/2010/03/03/feantsa-position-how-to-measure-and-monitor-homelessness-at-eu-level?bcParent=27 (Accessed November 15, 2025).

[42] Ouzzani M Hammady H Fedorowicz Z Elmagarmid A . Rayyan—A Web and Mobile App for Systematic Reviews. Syst Rev (2016) 5(1):1–10. 10.1186/s13643-016-0384-4 27919275 PMC5139140

[43] Hong Q Pluye P Fàbregues S Bartlett G Boardman F Cargo M Mixed Methods Appraisal Tool (MMAT), Version 2018. In: Canadian Intellectual Property Office, Industry Canada; #1148552 (2018). Available online at: http://mixedmethodsappraisaltoolpublic.pbworks.com/w/file/fetch/127916259/MMAT_2018_criteria-manual_2018-08-01_ENG.pdf (Accessed April 7, 2025).

[44] Hong QN Gonzalez-Reyes A Pluye P . Improving the Usefulness of a Tool for Appraising the Quality of Qualitative, Quantitative and Mixed Methods Studies, the Mixed Methods Appraisal Tool (MMAT). J Eval Clin Pract (2018) 24(3):459–67. 10.1111/jep.12884 29464873

[45] Souto RQ Khanassov V Hong QN Bush PL Vedel I Pluye P . Systematic Mixed Studies Reviews: Updating Results on the Reliability and Efficiency of the Mixed Methods Appraisal Tool. Int J Nurs Stud (2015) 52(1):500–1. 10.1016/j.ijnurstu.2014.08.010 25241931

[46] Hong QN Pluye P Fàbregues S Bartlett G Boardman F Cargo M Improving the Content Validity of the Mixed Methods Appraisal Tool: A Modified e-Delphi Study. J Clin Epidemiol (2019) 111:49–59. 10.1016/j.jclinepi.2019.03.008 30905698

[47] Hong QN Pluye P Fàbregues S Bartlett G Boardman F Cargo M Reporting the Results of the MMAT (Version 2018) (2020). Available online at: http://mixedmethodsappraisaltoolpublic.pbworks.com/w/file/140056890/Reporting%20the%20results%20of%20the%20MMAT.pdf (Accessed April 7, 2025).

[48] Ports KA Rostad WL Luo F Putnam M Zurick E . The Impact of the Low-Income Housing Tax Credit on Children’s Health and Wellbeing in Georgia. Child Youth Serv Rev (2018) 93:390–6. 10.1016/j.childyouth.2018.08.012 30613120 PMC6314036

[49] Boudreaux M Fenelon A Slopen N Newman SJ . Association of Childhood Asthma with Federal Rental Assistance. JAMA Pediatr (2020) 174(6):592–8. 10.1001/jamapediatrics.2019.6242 32150240 PMC7063540

[50] Coley RL Leventhal T Lynch AD Kull M . Relations Between Housing Characteristics and the Well-Being Of Low-Income Children and Adolescents. Dev Psychol (2013) 49(9):1775–89. 10.1037/a0031033 23244408 PMC3766502

[51] Currie J Yelowitz A . Are Public Housing Projects Good for Kids? J Public Econ (2000) 75(1):99–124. 10.1016/S0047-2727(99)00065-1

[52] Fenelon A . Does Public Housing Increase the Risk of Child Health Problems? Evidence from Linked Survey-Administrative Data. Hous Policy Debate (2022) 32(3):491–505. 10.1080/10511482.2021.1905027 35832732 PMC9272982

[53] Fenelon A Boudreaux M Slopen N Newman SJ . The Benefits of Rental Assistance for Children’s Health and School Attendance in the United States. Demography (2021) 58(4):1171–95. 10.1215/00703370-9305166 33970240 PMC8561436

[54] Fenelon A Slopen N Boudreaux M Newman SJ . The Impact of Housing Assistance on the Mental Health of Children in the United States. J Health Soc Behav (2018) 59(3):447–63. 10.1177/0022146518792286 30066591 PMC6657339

[55] Gensheimer SG Eisenberg MD Hindman D Wu AW Pollack CE . Examining Health Care Access and Health of Children Living in Homes Subsidized by the Low-Income Housing Tax Credit. Health Aff (2022) 41(6):883–92. 10.1377/hlthaff.2021.01806 35666971 PMC9379819

[56] Jacob BA Kapustin M Ludwig J . The Impact of Housing Assistance on Child Outcomes: Evidence from a Randomized Housing Lottery. Q J Econ (2015) 130(1):465–506. 10.1093/qje/qju030

[57] Leech TGJ . Subsidized Housing, Public Housing, and Adolescent Violence and Substance Use. Youth Soc (2012) 44(2):217–35. 10.1177/0044118X10388821

[58] Liaw EW . The Impact of Place-Based Housing Access on Academic Performance. In: Essays on Low-Income Housing Policies [Doctoral thesis]. San Diego: University of California (2023). p. 1–60. Available online at: https://escholarship.org/uc/item/9hj753dv (Accessed May 7, 2025).

[59] Meyers A Frank DA Roos N Peterson KE Casey VA Cupples LA Housing Subsidies and Pediatric Undernutrition. Arch Pediatr Adolesc Med (1995) 149(10):1079–84. 10.1001/archpedi.1995.02170230033004 7550809

[60] Meyers A Cutts D Frank DA Levenson S Skalicky A Heeren T Subsidized Housing and Children’s Nutritional Status Data from a Multisite Surveillance Study. Arch Pediatr Adolesc Med (2005) 159(6):551–6. 10.1001/archpedi.159.6.551 15939854

[61] Musa GJ Cheslack-Postava K Svob C Hernández D Tang H Duque-Villa Y Mental Health of High-Risk Urban Youth: The Housing Subsidies Paradox. Race Soc Probl (2021) 13(1):22–33. 10.1007/s12552-021-09322-7 34149954 PMC8211093

[62] Newman S Harkness J . Assisted Housing and the Educational Attainment of Children. J Hous Econ (2000) 9(1–2):40–63. 10.1006/jhec.2000.0259

[63] Sandel M Cook J Poblacion A Sheward R Coleman S Viveiros J Housing as a Health Care Investment: Affordable Housing Supports Children’s Health. Boston (2016). Available online at: https://childrenshealthwatch.org/housing-as-a-health-care-investment-affordable-housing-supports-childrens-health/ (Accessed May 7, 2025).

[64] Schwartz AE Horn KM Ellen IG Cordes SA . In: Do Housing Vouchers Improve Academic Performance? Evidence from New York City. Hoboken: Wiley (2019). p. 1–28. 10.1002/pam.22183

[65] Associates A Mills G Gubits D Orr L Long D Feins J Effects of Housing Vouchers on Welfare Families (2006). Available online at: https://www.huduser.gov/portal/publications/commdevl/hsgvouchers.html (Accessed April 5, 2025).

[66] Bovell-Ammon A Mansilla C Poblacion A Rateau L Heeren T Cook JT Housing Intervention for Medically Complex Families Associated with Improved Family Health: Pilot Randomized Trial. Health Aff (2020) 39(4):613–21. 10.1377/hlthaff.2019.01569 32250672

[67] Herzberg MP , Homework Starts with Home Research Partnership. Effects of Housing Subsidies and Community Social Support on School Attendance [Minn-LInK Brief No. 47]. Minneapolis: Center for Advanced Studies in Child Welfare, University of Minnesota (2022). Available online at: https://cascw.umn.edu/minn-link-brief-47-effects-housing-subsidies-and-community-social-support-school-attendance (Accessed April 5, 2025).

[68] Newman S Holupka CS . The Effects of Assisted Housing on Child Well-Being. Am J Community Psychol (2016) 60:1–13. 10.1002/ajcp.12100 27861993

[69] Rosero JA . The ABC of Housing Strategies: Are Housing Assistance Programs Effective in Enhancing Children’s well-being? In: On the importance of families and public policies for child development outcomes [Doctoral thesis]. Amsterdam: University of Amsterdam (2012). p. 63–94. Available online at: https://hdl.handle.net/11245/1.376640 (Accessed May 7, 2025).

[70] March EL De Cuba SE Gayman A Cook J Frank DA Meyers A Rx for Hunger: Affordable Housing. Boston (2009). Available online at: https://childrenshealthwatch.org/rx-for-hunger-affordable-housing/ (Accessed April 5, 2025).

[71] Pergamit M Cunningham M Hanson D . The Impact of Family Unification Housing Vouchers on Child Welfare Outcomes. Am J Community Psychol (2017) 60:1–11. 10.1002/ajcp.12136 28338225

[72] Turcotte DA Chaves E Gore R Adejumo KL Woskie S . The Impact of Housing Type on Low-Income Asthmatic Children Receiving Multifaceted Home Interventions. Public Health (2018) 164:107–14. 10.1016/j.puhe.2018.08.004 30266034

[73] Meyers A Rubin D Napoleone M Nichols K . Public Housing Subsidies May Improve Poor Children’s Nutrition. Am J Public Health (1993) 83(1):115. 10.2105/ajph.83.1.115 8417595 PMC1694511

[74] Gold S . Does Public Housing Reduce Housing Cost Burden Among Low-Income Families with Children? J Child Poverty (2020) 26(1):1–21. 10.1080/10796126.2019.1682754 32616994 PMC7331941

[75] Gold S . Housing Assistance and Residential Stability Among Low-Income Children. SSR (2018) 92(2):171–201. 10.1086/697372

[76] Israel BA Schulz AJ Parker EA Becker AB . Review of Community-Based Research: Assessing Partnership Approaches to Improve Public Health. Annu Rev Public Health (1998) 19:173–202. 10.1146/annurev.publhealth.19.1.173 9611617

[77] Lubell J . Reviewing State Housing Policy with a Child-Centered Lens: Opportunities for Engagement and Intervention. Washington DC (2013). Available online at: https://www.aecf.org/resources/reviewing-state-housing-policy-with-a-child-centered-lens (Accessed May 7, 2025).

[78] Hilber CAL Schöni O . Housing Policy and Affordable Housing. In: Centre for Economic Performance (CEP Occasional Papers 56) (2022). Available online at: https://ideas.repec.org/p/cep/cepops/56.html (Accessed May 7, 2025).

[79] Rajasekaran P Treskon M Greene S . Rent Control: What Does the Research Tell Us About the Effectiveness of Local Action? Washington DC (2019). Available online at: https://www.urban.org/research/publication/rent-control-what-does-research-tell-us-about-effectiveness-local-action (Accessed November 19, 2025).

[80] Sun W Zheng S Geltner DM Wang R . The Housing Market Effects of Local Home Purchase Restrictions: Evidence from Beijing. J Real Estate Fin Econ (2017) 55(3):288–312. 10.1007/s11146-016-9586-8

[81] Beckfield J Bambra C . Shorter Lives in Stingier States: Social Policy Shortcomings Help Explain the US Mortality Disadvantage. Soc Sci Med (2016) 171:30–8. 10.1016/j.socscimed.2016.10.017 27865604

[82] Reeves A McKee M Gunnell D Chang SS Basu S Barr B Economic Shocks, Resilience, and Male Suicides in the Great Recession: Cross-National Analysis of 20 EU Countries. Eur J Public Health (2015) 25(3):404–9. 10.1093/eurpub/cku168 25287115

[83] Sanbonmatsu L Katz LF Ludwig J Gennetian LA Duncan GJ Kessler RC Moving to Opportunity for Fair Housing Demonstration Program. Final Impacts Eval (2011). Available online at: https://www.huduser.gov/PORTAL/publications/pubasst/MTOFHD.html (Accessed April 28, 2025).

[84] Chetty R Hendren N Katz LF . The Effects of Exposure to Better Neighborhoods on Children: New Evidence from the Moving to Opportunity Experiment. AER (2016) 106(4):855–902. 10.1257/aer.20150572 29546974

[85] Keene DE Geronimus AT . Community-Based Support Among African American Public Housing Residents. J Urban Health (2011) 88(1):41–53. 10.1007/s11524-010-9511-z 21279452 PMC3042090

[86] Geronimus AT Thompson JP . To Denigrate, Ignore, or Disrupt: Racial Inequality in Health and the Impact of a Policy-Induced Breakdown of African American Communities. DBR (2004) 1(2):247–79. 10.1017/S1742058X04042031

[87] Chen KL Miake-Lye IM Begashaw MM Zimmerman FJ Larkin J McGrath EL Association of Promoting Housing Affordability and Stability with Improved Health Outcomes: A Systematic Review. JAMA Netw Open (2022) 5(11):e2239860. 10.1001/jamanetworkopen.2022.39860 36322083 PMC9631101

[88] Esping-Andersen G . The Three Worlds of Welfare Capitalism. Princeton: Princeton University Press (1990).

[89] Kemeny J . Comparative Housing and Welfare: Theorising the Relationship. J Hous Built Environ (2001) 16(1):53–70. 10.1023/a:1011526416064

[90] Goetz EG . New Deal Ruins: Race, Economic Justice, and Public Housing Policy. Ithaca: Cornell University Press (2013). Available online at: http://www.jstor.org/stable/10.7591/j.ctt1xx4hq (Accessed April 28, 2025).

[91] Hackworth J . The Neoliberal City: Governance, Ideology, and Development in American Urbanism. Ithaca: Cornell University Press (2007). Available online at: http://www.jstor.org/stable/10.7591/j.ctt7z5hr (Accessed April 28, 2025).

[92] Kemeny J . From Public Housing to the Social Market: Rental Policy Strategies in Comparative Perspective. 1st ed. London: Routledge (2002). p. 211. Available online at: https://www.routledge.com/From-Public-Housing-Soc-Market/Kemeny/p/book/9780415083652 (Accessed April 26, 2025).

