# Supplementary material for: Interventions to Mitigate the Effects of Housing Insecurity on Child and Adolescent Health: A Scoping Review
Source: Public Health Rev. 2026 Feb 6;46:1609177. doi: 10.3389/phrs.2025.1609177 (PMC12920252; doi:10.3389/phrs.2025.1609177)
Supplement: Supplementary file 1 [file Table1.docx]

***Supplementary Table 1.*** *Search strategies developed for each database or search engine (Spain, 2025).*

| **Database** | **Search strategy** |
| --- | --- |
| PubMed | ("housing"[Title/Abstract] OR "housing instability"[Title/Abstract] OR "unstable housing"[Title/Abstract] OR "insecure housing"[Title/Abstract] OR "housing insecurity"[Title/Abstract] OR "housing stability"[Title/Abstract] OR "unaffordable housing"[Title/Abstract] OR "housing affordability"[Title/Abstract] OR "evict*"[Title/Abstract] OR "rent arrear*"[Title/Abstract] OR "mortgage foreclosure*"[Title/Abstract] OR “housing displace*”[Title/Abstract] OR “doubling-up”[Title/Abstract] OR “overcrowd*”[Title/Abstract] OR “housing mobility”[Title/Abstract] OR “rent*”[Title/Abstract] OR “tenant*”[Title/Abstract] OR “dweller*”[Title/Abstract] OR “leaseholder*”[Title/Abstract]) AND ("intervention"[Title/Abstract] OR "child welfare"[Title/Abstract] OR "pilot projects"[Title/Abstract] OR "public policy"[Title/Abstract] OR "housing voucher*"[Title/Abstract] OR "housing subsid*"[Title/Abstract] OR "housing assistance"[Title/Abstract] OR "initiative"[Title/Abstract] OR "public housing"[Title/Abstract] OR “housing policy”[Title/Abstract]) AND ("child"[Title/Abstract] OR "adolescent"[Title/Abstract] OR "child*"[Title/Abstract] OR "preschool child"[Title/Abstract] OR "infant"[Title/Abstract] OR “kid”[Title/Abstract] OR “youth”[Title/Abstract] OR “baby”[Title/Abstract] OR “pediatric*”[Title/Abstract] OR “newborn”[Title/Abstract] OR “toddler”[Title/Abstract] OR “preteen”[Title/Abstract]) |
| Web of Science | TS=("housing instability" OR "unstable housing" OR "insecure housing" OR "housing insecurity" OR "housing stability" OR "unaffordable housing" OR "housing affordability" OR "evict*" OR "rent arrear*" OR "mortgage foreclosure*" OR "housing displace*" OR "doubling-up" OR "overcrowd*" OR "housing mobility" OR "rent*" OR "tenant*" OR "dweller*" OR "leaseholder*") AND TS=("intervention" OR "child welfare" OR "pilot projects" OR "public policy" OR "housing voucher*" OR "housing subsid*" OR "housing assistance" OR "initiative" OR "public housing" OR "housing policy") AND TS=("child" OR "adolescent" OR "child*" OR "preschool child" OR "infant" OR "kid" OR "youth" OR "baby" OR "pediatric*" OR "newborn" OR "toddler" OR "preteen") |
| Scopus | TITLE-ABS-KEY ( "housing instability" OR "unstable housing" OR "insecure housing" OR "housing insecurity" OR "housing stability" OR "unaffordable housing" OR "housing affordability" OR "evict*" OR "rent arrear*" OR "mortgage foreclosure*" OR "housing displace*" OR "doubling-up" OR "overcrowd*" OR "housing mobility" OR "tenant*" OR "dweller*" OR "leaseholder*" ) AND TITLE-ABS-KEY ( "intervention" OR "child welfare" OR "pilot projects" OR "public policy" OR "housing voucher*" OR "housing subsid*" OR "housing assistance" OR "initiative" OR "public housing" OR "housing policy" ) AND TITLE-ABS-KEY ( "child" OR "adolescent" OR "child*" OR "preschool child" OR "infant" OR "kid" OR "pediatric*" OR "newborn" OR "toddler" ) |
| CINAHL | AB (housing instability OR unstable housing OR insecure housing OR housing insecurity OR housing stability OR unaffordable housing OR housing affordability OR eviction OR rent arrear* OR mortgage foreclosure* OR housing displace* OR doubling-up OR overcrowd* OR housing mobility) AND AB (intervention OR child welfare OR pilot projects OR public policy OR housing voucher* OR housing subsid* OR housing assistance OR initiative OR public housing OR housing policy) AND AB (child OR adolescent OR child* OR preschool child OR infant) |
| Carrot2* | 1. housing intervention policy child  2. housing instability intervention policy child  3. housing insecurity intervention policy child  4. unaffordable housing intervention policy child  5. eviction intervention policy child  6. housing intervention policy adolescent  7. housing instability intervention policy adolescent  8. housing insecurity intervention policy adolescent  9. unaffordable housing intervention policy adolescent  10. eviction intervention policy adolescent |

*Due to the way this search engine works, we conducted ten different searches by combining various keywords.
