# Supplementary material for: Interventions to Mitigate the Effects of Housing Insecurity on Child and Adolescent Health: A Scoping Review
Source: Public Health Rev. 2026 Feb 6;46:1609177. doi: 10.3389/phrs.2025.1609177 (PMC12920252; doi:10.3389/phrs.2025.1609177)
Supplement: Supplementary file 2 [file Table2.docx]

**Supplementary Table 2.** Overview of study characteristics and main results of included interventions (Spain, 2025).

| **Reference and document type** | **Aim** | **Intervention characteristics** | **Intervention description** | **Methodology** | **Instruments** | **Study quality score** | **Main results** |
| --- | --- | --- | --- | --- | --- | --- | --- |
| Abt Associates et al., 2006 [65] (Report) | To evaluate the impacts of the Welfare to Work Voucher (WtWV) program on low-income families, particularly focusing on housing quality, neighborhood environment, employment, earnings, public assistance receipt, and family well-being, including child well-being. | **Country:** USA  **CAA age:** 5-15 years  **Level of intervention:** Intermediate | **Housing vouchers:** Provision of a housing voucher through the Welfare-to-Work Voucher (WtWV) program that subsidizes monthly rent payments, with the subsidy amount equal to the payment standard set by the housing authority minus 30% of the family's adjusted monthly income. Allows families to choose and rent housing in the private market. | **Mixed methods** (randomized controlled trial + semi-structured interviews)  **N:** IG n= ~2.050 (receiving housing vouchers); CG n= ~2.050 (not receiving housing vouchers)  **Number of interventions studied:** 1  **Period covered by analysis:** 1999-2004 | **Survey:** baseline and follow-up  **Administrative records:** HUD housing assistance data  **Population census:** neighborhood data  **Interviews:** qualitative data gathering | 80% | **MENTAL HEALTH**  **Behavior:** No significant effects on measures of child behavior.  **EDUCATIONAL OUTCOMES**  **School attendance:** Reduced likelihood of children (intervention group) being out of school due to health, financial, or disciplinary problems, for girls and children under 6 at baseline.  **Short-term academic metrics:** Increased likelihood of children (intervention group) repeating a grade.  **Long-term educational attainment:** No significant effects on educational attainment or college enrollment. |
| Boudreaux et al., 2020 [49] (Peer-reviewed article) | To examine whether participation in rental assistance programs is associated with child asthma. | **Country:** USA  **CAA age:** 0-17 years  **Level of intervention:** Structural, Intermediate | **Public housing:** Government-owned buildings in which all units are subsidized, tenants typically pay 30% of their monthly income in rent, and eligibility and allocation are determined by local housing authorities based on waiting lists and priority criteria.  **Housing vouchers:** The Housing Choice Voucher (HCV) program provides direct rental subsidies to families, allowing them to choose and rent housing in the private market. Families contribute with approximately 30% of their monthly income.  **Subsidies to private developers of affordable housing:** HUD’s multifamily housing, private landlords offer a limited number of subsidized units at reduced rents, with tenants paying about 30% of their income and federal funds covering the remainder. | **Quantitative** (cross-sectional with comparison group)  **N:** IG n=2022 (receiving housing assistance); CG n=970 (not receiving assistance)  **Number of interventions studied:** 3  **Period covered by analysis:** 1999-2014 | **Survey:** Survey of Income and Program Participation (SIPP) from 1992 and 1993; Current Population Survey (CPS) from 1990 and 1995. | 100% | **PHYSICAL HEALTH**  **Health conditions:** No significant changes in the incidence of asthma attacks.  **HEALTH CARE ACCES AND UTILIZATION**  **Urgency care:** CAA that had suffered an asthmatic crisis in the last year and were in rental assistance projects experienced an 18% reduction in the use of emergency services by asthma. Especially marked reduction in public or multi-family housing (36.6%). |
| Bovell-Amon et al., 2020 [66] (Peer-reviewed article) | Analyze whether the intervention "Housing Prescriptions as Health Care" —which combines priority affordable housing, case management, financial and legal services— improves the physical and mental health of "medically complex" families (with adult members or CAA with chronic illnesses, disabilities or intensive use of health services), compared to standard care (guided hospital resources and social services). | **Country:** USA  **CAA age:** 0-11 years  **Level of intervention:** Intermediate-Individual/group | **Multi-assistance (legal, financial, medical, housing, and wraparound support):** “Housing Prescriptions as Healthcare” is a personalized program for families facing homelessness or housing instability, linking hospitals, social services, and local government. It combines expedited public housing, case management, legal and financial support, and medical documentation to secure stable housing as a critical part of care. | **Quantitative** (longitudinal with comparison group)  **N:** IG n=36 (families receiving intervention); CG n=31 (families receiving standard care)  **Number of interventions studied:** 1  **Period covered by analysis:** 2016-2019 | **Validated questionnaires:** General health status questionnaire (NHANES); mental health scales (GAD-2 and PHQ-2); Parent's Evaluation of Developmental Status (PEDS).  **Medical records:** electronic data to obtain the BMI and the number of urgency visits/hospitalizations. | 80% | **GENERAL HEALTH STATUS:** Decrease in the percentage of children with fair/poor health: 32 percentage points more in IG compared to CG.  **PHYSICAL HEALTH**  **Health conditions:** Children from families who accessed housing (11 cases) showed no development delay (0% vs. 62.5% in non-hosted), although the sample was small. There were no significant differences in development risk (PEDS) between IG and CG.  **Nutrition/Growth:** No statistically significant changes were observed in children’s weight or nutrition.  **HEALTH CARE ACCESS AND UTILIZATION**  **Urgency care:** Urgency care visits decreased in both groups.  **Hospitalizations:** Hospitalizations decreased in both groups. |
| Coley et al., 2013 [50] (Peer-reviewed article) | To comprehensively assess the impact of multiple housing characteristics (quality, stability, type, and cost) on the well-being of low-income children and adolescents, examining cognitive, emotional, and behavioral outcomes. | **Country:** USA  **CAA age:** 2-21 years  **Level of intervention:** Structural, Intermediate | **Public housing:** Government-owned buildings in which all units are subsidized, tenants typically pay 30% of their monthly income in rent, and eligibility and allocation are determined by local housing authorities based on waiting lists and priority criteria.  **Housing vouchers:** The Housing Choice Voucher (HCV) program provides direct rental subsidies to families, allowing them to choose and rent housing in the private market. Families contribute with approximately 30% of their monthly income. | **Quantitative** (longitudinal with comparison group)  **N:** IG n=1.194 (living in public housing or housing vouchers); CG1 n=682 (living in private rent); CG2 n=585 (living in property)  **Number of interventions studied:** 2  **Period covered by analysis:** 1999-2005 | **Survey:** quality, stability, type and cost of housing.  **Validated questionnaires:** Child Behavior Checklist (CBCL) for emotional problems and Woodcock-Johnson Subtests (reading and mathematics, cognitive skills). | 100% | **MENTAL HEALTH**  **Psychological distress:** Adolescents in subsidized housing reported less anxiety/depression symptoms than those in private rent (associated with lower family stress). Slower growth of internalizing problems with age (compared to private renting). Little children (2-9 years) did not benefit from this.  **Behavior:** No significant effects on externalizing problems.  **EDUCATIONAL OUTCOMES**  **Short-term academic metrics:** Better math skills in teenagers from the intervention group. No significant effect for little kids (2-9 years). |
| Currie & Yelowitz, 2000 [51] (Peer-reviewed article) | To evaluate the impact of participation in public housing projects on the quality of housing and on the educational performance of children in low-income families. | **Country:** USA  **CAA age:** 0-17 years  **Level of intervention:** Structural | **Public housing:** Government-owned buildings in which all units are subsidized, tenants typically pay 30% of their monthly income in rent, and eligibility and allocation are determined by local housing authorities based on waiting lists and priority criteria. | **Quantitative** (cross-sectional with comparison group)  **N:** IG n=1.048 (families living in public housing); CG n=20.670 (families not living in public housing)  **Number of interventions studied:** 1  **Period covered by analysis:** 1990-1995 | **Survey:** Survey of Income and Program Participation (SIPP) of 1992 and 1993; Current Population Survey (CPS) of March 1990 and 1995 (educational outcomes obtained from 1990). | 100% | **EDUCATIONAL OUTCOMES**  **Short-term academic metrics:** CAA in public housing projects were 11% less likely to repeat course (vs. CAA out of projects) after being controlled by sociodemographic factors.  The effect was strongest on african-american CAA, where the reduction was 19%, while on white CAA it was not statistically significant. The reduction in boys living in public housing was 18%, while in girls it was not statistically significant. |
| Fenelon, 2022 [52] (Peer-reviewed article) | To examine the effects of living in public housing on the risk of health problems (including frequent diarrhea, frequent headaches, skin allergies, asthma and good/bad health status) among children. | **Country:** USA  **CAA age:** 0-17 years  **Level of intervention:** Structural | **Public housing:** Government-owned buildings in which all units are subsidized, tenants typically pay 30% of their monthly income in rent, and eligibility and allocation are determined by local housing authorities based on waiting lists and priority criteria. | **Quantitative** (longitudinal with comparison group)  **N:** IG n=331 (living in public housing); CG1 n=145 (not living in public housing); CG2 n=8.873 (not living in public housing and income below 200% of the poverty line); CG3 n=33.539 (in wait-list for public housing)  **Number of interventions studied:** 1  **Period covered by analysis:** 1999-2014 | **Survey:** National Health Interview Survey (NHIS)  **Administrative records:** HUD housing assistance records | 100% | **PHYSICAL HEALTH**  **Health conditions:** 39% of children in public housing have at least one health problem, compared to 26% of those who do not live there and 31% of low-income children who live on rent. However, this percentage is almost equal to 38% of the children who will enter next month. |
| Fenelon et al., 2021 [53] (Peer-reviewed article) | To evaluate whether access to housing aid (public housing, multi-family housing and HCV) reduces school days lost due to illness in children from low-income families. | **Country:** USA  **CAA age:** 5-17 years  **Level of intervention:** Structural, Intermediate | **Public housing:** Government-owned buildings in which all units are subsidized, tenants typically pay 30% of their monthly income in rent, and eligibility and allocation are determined by local housing authorities based on waiting lists and priority criteria.  **Housing vouchers:** The Housing Choice Voucher (HCV) program provides direct rental subsidies to families, allowing them to choose and rent housing in the private market. Families contribute with approximately 30% of their monthly income.  **Subsidies to private developers of affordable housing:** HUD’s multifamily housing, private landlords offer a limited number of subsidized units at reduced rents, with tenants paying about 30% of their income and federal funds covering the remainder. | **Quantitative** (longitudinal with comparison group)  **N:** IG n=1.485 (receiving housing assistance); CG n=571 (in a housing assistance wait-list)  **Number of interventions studied:** 3  **Period covered by analysis:** 1999-2014 | **Survey:** National Health Interview Survey (NHIS)  **Administrative records:** HUD’s housing assistance data | 100% | **PHYSICAL HEALTH**  **Health conditions:** CAA in intervention group had a lower risk of ear infection.  **HEALTHCARE ACCES AND UTILIZATION**  **Urgency care:** CAA in intervention group had fewer asthma urgency care visits.  **Hospitalizations:** CAA in intervention group had fewer hospitalizations.  **EDUCATIONAL OUTCOMES**  **School attendance:** CAA in intervention group were 22% less days off school than those in the comparison group, with a stronger effect on adolescents (12-17 years) than on young children (5-11 years).  All health benefits were specially present on CAA in housing vouchers. No significant effects reported for non-hispanic black CAA. |
| Fenelon et al., 2018 [54] (Peer-reviewed article) | To examine the impact of the USA federal housing assistance programs (public housing, housing choice vouchers, and multifamily housing) on the mental health of children aged 2 to 17. | **Country:** USA  **CAA age:** 2-17 years  **Level of intervention:** Structural, Intermediate | **Public housing:** Government-owned buildings in which all units are subsidized, tenants typically pay 30% of their monthly income in rent, and eligibility and allocation are determined by local housing authorities based on waiting lists and priority criteria.  **Housing vouchers:** The Housing Choice Voucher (HCV) program provides direct rental subsidies to families, allowing them to choose and rent housing in the private market. Families contribute with approximately 30% of their monthly income.  **Subsidies to private developers of affordable housing:** HUD’s multifamily housing, private landlords offer a limited number of subsidized units at reduced rents, with tenants paying about 30% of their income and federal funds covering the remainder. | **Quantitative** (longitudinal with comparison group)  **N:** IG n=1.536 (receiving housing assistance): CG n=431 (on a wait-list for housing assistance)  **Number of interventions studied:** 3  **Period covered by analysis:** 1999-2014 | **Survey:** National Health Interview Survey (NHIS), specifically Strengths and Difficulties Questionnaire (SQD), a patient-reported tool for mental health; American Community Survey (ACS)  **Administrative records:** HUD’s housing assistance data  **Population census:** neighborhood data | 100% | **MENTAL HEALTH**  **Psychological distress:** Mental health improvement; CAA in public housing had 1,38 less points at the SQD symptom scale.  **Behavior:** CAA in public housing were 80% less likely to have a socio-emotional problem and 72% less likely to experience emotional difficulties.  No reported health benefits for children 2-5 years and for CAA in multifamily housing and housing vouchers. |
| Gensheimer et al., 2022 [55] (Peer-reviewed article) | To examine whether access to health services and health status among children living in Low-Income Housing Tax Credit (LIHTC) homes are better than those of low-income children who do not live in this type of housing. | **Country:** USA  **CAA age:** 0-17 years  **Level of intervention:** Intermediate | **Subsidies to private developers of affordable housing:** Low-Income Housing Tax Credit (LIHTC); the program incentivizes developers to build rental housing for low-income households by offering tax credits. Tenants typically pay 30% of either 50% or 60% of the Area Median Income (AMI), meaning rent is based on set income thresholds rather than the tenant’s actual income. However, Housing Choice Vouchers can be used to help cover rent in LIHTC units. These developments are often concentrated in high-poverty neighborhoods. | **Quantitative** (cross-sectional with comparison group)  **N:** IG n=746 (living in LIHTC dwellings); CG n=63.840 (low-income not living in LIHTC dwellings)  **Number of interventions studied:** 1  **Period covered by analysis:** 2004-2016 | **Survey:** National Health Interview Survey (NHIS) from 2004 and 2016  **Administrative records:** LIHTC user’s records | 100% | **PHYSICAL HEALTH**  **Health conditions:** CAA in LIHTC have higher chances (3.0 percent more) of currently having asthma.  **HEALTHCARE ACCES AND UTILIZATION**  **Preventive care:** CAA living in LIHTC dwellings are 3.8 percent more likely to have made a pediatric control visit in the last 12 months and 4.8 percentage points more likely to have visited the dentist in the last 6 months compared to low-income children who did not live on LIHTC properties.  **EDUCATIONAL OUTCOMES**  **School attendance:** CAA in LIHTC have higher chances (3.5 percent more) of chronic school absenteeism. |
| Herzberg et al., 2022 [67] (Report) | To examine the relationship between community-level social supports (specifically, place-based programs like Promise Neighborhoods) and housing subsidies (through the Homework Starts with Home pilot program) on school attendance among children experiencing homelessness or housing instability. | **Country:** USA  **CAA age:** 13-14 years  **Level of intervention:** Intermediate-Individual/group | **Multi-assistance (legal, financial, medical, housing, and wraparound support):** “Homework Starts With Home” (HSWH); grant program focused on addressing homelessness and housing instability among students and their families. Funding may be used to provide supportive services to find and keep housing or financial help for rent deposit, rent assistance, utilities, housing application assistance and other housing related costs. “Promise Neighborhoods”; US federal programs that aim to improve academic results and the well-being of children in disadvantaged communities. They are based on a comprehensive support model (or wrap-around), which combines education (schools with additional resources), health (access to medical services), family support (advice, training, financial aid) and community networks (collaboration with local organizations). | **Quantitative** (longitudinal with comparison group)  **N:** IG1 n=87 (receiving both interventions); IG2 n=132 (only receiving rental assistance); CG n=145 (not receiving any intervention)  **Number of interventions studied:** 2  **Period covered by analysis:** 2013-2017 | **Administrative records:** Minnesota Housing Finance Agency; Minnesota Homeless Management Information System  **Educational records:** Minnesota Automated Reporting Student System | 80% | **EDUCATIONAL OUTCOMES**  **School attendance:** CAA in Promise Neighborhood schools maintained high attendance (91–92%) during the first two years, but showed no further improvement. CAA who received rental assistance through HSWH but did not attend a Promise Neighborhood school improved attendance by 1% per year (equivalent to +1.5 days/year).  The combination of rental assistance and attending a Promise Neighborhood school did not show additional benefits compared to receiving just one of the two supports. |
| Jacob et al., 2015 [56] (Peer-reviewed article) | To evaluate the impact of housing vouchers on the long-term results of children, including education (secondary qualifications and graduation), health (visits to emergencies or hospitalisations) and juvenile police arrests. | **Country:** USA  **CAA age:** Unspecified  **Level of intervention:** Intermediate | **Housing vouchers:** The Housing Choice Voucher (HCV) program provides direct rental subsidies to families, allowing them to choose and rent housing in the private market. Families contribute with approximately 30% of their monthly income. | **Quantitative** (longitudinal with comparison group)  **N:** IG n=18.347 (receiving housing vouchers); CG n=48.263 (not receiving housing vouchers but eligible for it)  **Number of interventions studied:** 1  **Period covered by analysis:** 1997-2011 | **Educational records:** Chicago Public Schools (attendance qualifications, graduation)  **Medical records:** Medicaid (visits to urgency care or hospitalizations)  **Legal records:** State police (police arrests of adolescents)  **Population census:** Local changes at the geographical level | 100% | **HEALTHCARE ACCES AND UTILIZATION**  **Urgency care:** No significant results.  **Hospitalizations:** No significant results.  **EDUCATIONAL OUTCOMES**  **Short-term academic metrics:** Non-significant differences of 0.01-0.06 standard deviations in grades.  **Long-term educational attainment:** Non-significant increase of 1.5-2.9 percentage points in secondary completion. |
| Leech et al., 2012 [57] (Peer-reviewed article) | To examine the separate relationships between public housing residence and subsidized housing residence on adolescent health risk behaviors, specifically violence and substance use. | **Country:** USA  **CAA age:** 14-19 years  **Level of intervention:** Structural, Intermediate | **Public housing:** Government-owned buildings in which all units are subsidized, tenants typically pay 30% of their monthly income in rent, and eligibility and allocation are determined by local housing authorities based on waiting lists and priority criteria.  **Housing vouchers:** The Housing Choice Voucher (HCV) program provides direct rental subsidies to families, allowing them to choose and rent housing in the private market. Families contribute with approximately 30% of their monthly income. | **Quantitative** (longitudinal with comparison group)  **N:** IG1 n=125 (living in public housing); IG2 n=90 (living in housing vouchers); CG n=2315 (not receiving housing assistance)  **Number of interventions studied:** 2  **Period covered by analysis:** 2002-2004 | **Survey:** National Longitudinal Survey of Youth for participant’s mothers (housing type, ethnicity, income, education, etc.); Self-report of adolescents (violence, drug use, etc.) | 100% | **MENTAL HEALTH**  **Behavior:** CAA in housing vouchers reported a significant decrease in substance use (-5%), and a downward trend in excessive alcohol/marijuana consumption (-8%, marginally significant). For CAA in public housing, no significant differences were found in any risk behavior compared to the control group, except for a tendency to increase excessive alcohol/marijuana consumption (not significant). |
| Liaw, 2023 [58] (Doctoral thesis) | Analyze whether the transfer of school-age children to LIHTC-funded housing has positive results in terms of school performance (attendance, qualifications in English and mathematics, completion of secondary school and enrollment and completion of post-secondary education). | **Country:** USA  **CAA age:** 7-17 years  **Level of intervention:** Intermediate | **Subsidies to private developers of affordable housing:** Low-Income Housing Tax Credit (LIHTC); the program incentivizes developers to build rental housing for low-income households by offering tax credits. Tenants typically pay 30% of either 50% or 60% of the Area Median Income (AMI), meaning rent is based on set income thresholds rather than the tenant’s actual income. However, Housing Choice Vouchers can be used to help cover rent in LIHTC units. These developments are often concentrated in high-poverty neighborhoods. | **Quantitative** (longitudinal with comparison group)  **N:** IG n=4.546 (students who moved into LIHTC units); CG1 n=6.160 (matched students who never lived in LIHTC, including movers and non-movers); CG2 n=4.968 (matched students who moved but did not move into LIHTC units, with similar move/school change patterns)  **Number of interventions studied:** 1  **Period covered by analysis:** 2001-2013 | **Administrative records:** San Diego Unified School District database; LIHTC users database | 100% | **EDUCATIONAL OUTCOMES**  **School attendance:** Compared with the control group of CAA who do not move or move to housing without subsidizing, CAA who accessed LIHTC units experienced a decrease of 0.28 percentage points in school absenteeism (from 7-17 years). CAA who remained in the same school after the move to a LIHTC unit did not have changes in absenteeism. CAA who changed both residence and school showed a sharper reduction in absenteeism (–0.61 percentage points).  **Short-term academic metrics:** CAA who accessed LIHTC units experimented an increase of 0.049 standard deviations in English scores (7-17 years) and 0.048 standard deviations in mathematics (7-13 years), the latter with marginal statistical significance. Students who maintained the same school after moving to a LIHTC home experienced increases of 0.094 standard deviations in English and 0.096 in mathematics. Children who changed both residence and school had a deterioration in math grades (–0,123 standard deviations). |
| March et al., 2009 [70] (Report) | To investigate the link between affordable housing and childhood health, particularly focusing on how subsidized housing impacts food security, nutritional status, and overall well-being in low-income children. | **Country:** USA  **CAA age:** 0-3 years  **Level of intervention:** Structural, Intermediate | **Public housing:** Government-owned buildings in which all units are subsidized, tenants typically pay 30% of their monthly income in rent, and eligibility and allocation are determined by local housing authorities based on waiting lists and priority criteria.  **Housing vouchers:** The Housing Choice Voucher (HCV) program provides direct rental subsidies to families, allowing them to choose and rent housing in the private market. | **Quantitative** (cross-sectional with comparison group)  **N:** 36.000; IG n=unspecified (receiving housing assistance); CG n=unspecified (in a wait-list for receiving it)  **Number of interventions studied:** 2  **Period covered by analysis:** 1998-2008 | **Survey:** Socio-economic and food insecurity data  **Medical records:** Children’s weight and health status | 60% | **GENERAL HEALTH STATUS:** Children with housing assistance were 28% more likely to be classified as "healthy".  **PHYSICIAL HEALTH**  **Nutrition/Growth:** Children with housing assistance had a 35% lower risk of food insecurity. Subsidized housing has the greatest impact on protecting the growth and reducing underweight in children from food insecure households, with a 52% lower rate of underweight compared to those on the waitlist. |
| Meyers et al., 1993 [73] (Letter to the editor) | To evaluate whether public housing subsidies improve the nutritional status of children in low-income families, specifically in relation to iron deficiency. | **Country:** USA  **CAA age:** Unspecified.  **Level of intervention:** Structural | **Public housing:** Government-owned buildings in which all units are subsidized, tenants typically pay 30% of their monthly income in rent, and eligibility and allocation are determined by local housing authorities based on waiting lists and priority criteria. | **Quantitative** (retrospective with comparison group)  **N:** 580; IG n=unspecified (living in public housing); CG n=unspecified (not living in public housing)  **Number of interventions studied:** 1  **Period covered by analysis:** 1989-1990 | **Administrative records:** Demographic and housing data provided by the Boston Housing Authority and the Metropolitan Housing Authority.  **Medical records:** Data from the studied clinic. | 40% | **PHYSICAL HEALTH**  **Health conditions:** Iron deficiency was lower among children with housing subsidies (19% IG and 30% CG). Odds Ratios also indicate a marginally significant trend towards lower risk in the group with subsidies.  **Nutrition/Growth:** No significant metrics were found in height, weight, etc. |
| Meyers et al., 1995 [59] (Peer-reviewed article) | To test the hypothesis that receipt of housing subsidies by poor families is associated with improved nutritional status of their children, as measured by growth indicators. | **Country:** USA  **CAA age:** 0-3 years  **Level of intervention:** Structural, Intermediate | **Public housing:** Government-owned buildings in which all units are subsidized, tenants typically pay 30% of their monthly income in rent, and eligibility and allocation are determined by local housing authorities based on waiting lists and priority criteria.  **Housing vouchers:** The Housing Choice Voucher (HCV) program provides direct rental subsidies to families, allowing them to choose and rent housing in the private market. Families contribute with approximately 30% of their monthly income. | **Quantitative** (cross-sectional with comparison group)  **N:** IG n=42 (receiving housing assistance); CG1 n=51 (on wait-list for housing assistance); CG2 n=60 (no assistance and no wait-list)  **Number of interventions studied:** 2  **Period covered by analysis:** March-April 1992 | **Survey:** Questions about health care, social programs and housing  **Medical records:** Weight, height, and other indicators using data from the National Center for Health Statistics (NCHS), to see the proportion of children with low growth | 100% | **PHYSICAL HEALTH**  **Nutrition/Growth:** Children from families receiving housing subsidies had significantly higher z-scores for weight-for-age (P=0.03) and weight-for-height (P=0.04) compared to children on the waitlist. The risk of low growth indicators was 3.3% in the subsidized group, versus 21.6% in the waitlisted group (adjusted odds ratio = 8.2, P=0.002). Hispanic and white children showed significant differences in some indicators (e.g., Hispanics had higher weight-for-height z-scores). |
| Meyers et al., 2005 [60] (Peer-reviewed article) | To examine the relationship between receiving housing subsidies and the nutritional and health status of young children in low-income families, particularly those experiencing food insecurity. | **Country:** USA  **CAA age:** 0-3 years  **Level of intervention:** Structural, Intermediate | **Public housing:** Government-owned buildings in which all units are subsidized, tenants typically pay 30% of their monthly income in rent, and eligibility and allocation are determined by local housing authorities based on waiting lists and priority criteria.  **Housing vouchers:** The Housing Choice Voucher (HCV) program provides direct rental subsidies to families, allowing them to choose and rent housing in the private market. Families contribute with approximately 30% of their monthly income. | **Quantitative** (cross-sectional with comparison group)  **N:** IG n=3.217 (families receiving housing assistance); CG n=8.506 (families without housing assistance)  **Number of interventions studied:** 2  **Period covered by analysis:** 1998-2003 | **Survey:** Socio-economic data, participation in other social programs, and food security scale.  **Medical records:** Children’s weight, health status, hospitalizations, etc. | 100% | **GENERAL HEALTH STATUS:** No significant results found.  **PHYSICAL HEALTH**  **Nutrition/Growth:** Children from food-insecure families with housing subsidies had better weight-for-age (adjusted mean z-score: 0.205) compared to those without subsidies (-0.025; p < 0.001). This difference was not significant among food-secure families. Children from food-insecure families without subsidies were 2.11 times more likely (OR: 2.11; 95% CI: 1.34–3.32) to have a weight-for-age more than 2 SD below the mean.  **HEALTHCARE ACCES AND UTILIZATION**  **Hospitalizations:** No significant results found. |
| Musa et al., 2021 [61] (Peer-reviewed article) | To evaluate the association between the different types of housing subsidies (public housing, Housing Choice Vouchers, or both) and the mental health of adolescents, with special attention to symptoms and internalizing disorders (anxiety, depression) and externalizing (disruptive behavior). | **Country:** USA  **CAA age:** 9-17 years  **Level of intervention:** Structural, Intermediate | **Public housing:** Government-owned buildings in which all units are subsidized, tenants typically pay 30% of their monthly income in rent, and eligibility and allocation are determined by local housing authorities based on waiting lists and priority criteria.  **Housing vouchers:** The Housing Choice Voucher (HCV) program provides direct rental subsidies to families, allowing them to choose and rent housing in the private market. Families contribute with approximately 30% of their monthly income. | **Quantitative** (cross-sectional with comparison group)  **N:** IG1 n=185 (receiving public housing only); IG2 n=96 (receiving housing vouchers only); IG3 n=42 (receiving both public housing and housing vouchers); CG n=388 (no housing assistance)  **Number of interventions studied:** 2  **Period covered by analysis:** Unspecified. | **Validated questionnaires:** Diagnostic Interview Schedule for Children; Columbia Impairment Scale; Collective Efficacy Scale; Family Resource Scale  **Visual assessment:** Direct observation of cleanliness or structural defects of the house by interviewers | 100% | **MENTAL HEALTH**  **Psychological distress:** CAA in the intervention groups (especially those with both subsidies) show more symptoms of psychological distress (RR=1.30, 95% CI: 1.04–1.64) than those without subsidies, after adjusting for sociodemographic factors.  **Behavior:** CAA in the intervention groups had greater functional impairment, but the differences were not significant after adjusting for sociodemographic characteristics. Economic resources slightly attenuated the association with externalizing symptoms. |
| Newman & Harkness, 2000 [62] (Peer-reviewed article) | To examine the long-term effects of assisted housing (public housing and privately owned subsidized housing) on the educational attainment of children. | **Country:** USA  **CAA age:** Unspecified  **Level of intervention:** Structural, Intermediate | **Public housing:** Government-owned buildings in which all units are subsidized, tenants typically pay 30% of their monthly income in rent, and eligibility and allocation are determined by local housing authorities based on waiting lists and priority criteria.  **Subsidies to private developers of affordable housing:** Farmers Home Section 515, a Federal program aimed at rural areas that offers low-interest loans for the construction of affordable housing, and Low-Income Housing Tax Credit (LIHTC), a program that incentivizes developers to build rental housing for low-income households by offering tax credits. Tenants typically pay 30% of either 50% or 60% of the Area Median Income (AMI), meaning rent is based on set income thresholds rather than the tenant’s actual income. | **Quantitative** (longitudinal with comparison group)  **N:** IG1 n=135 (living in public housing); IG2 n=102 (subsidized private housing, i.e., state rental assistance programs of housing vouchers, Farmers Home Section 515 and LIHTC); CG n=712 (no housing assistance)  **Number of interventions studied:** 3  **Period covered by analysis:** 1967-1995 | **Survey:** Panel Study of Income Dynamics (PSID) for years of education, graduation, post-secondary education, etc.  **Administrative records:** HUD’s federal housing assistance data; States housing assistance programs data  **Population census:** Socio-demographic and poverty data | 100% | **EDUCATIONAL OUTCOMES**  **Long-term educational attainment:** For CAA in public housing, the negative differences in education (such as lower high school graduation rates) disappear after controlling for socioeconomic factors. There are no significant positive or negative effects in the instrumental variable models.  For CAA in subsidized private housing, there are neutral effects, similar to those of non-assisted individuals. No significant differences are found in educational outcomes for children living in large cities (used as a proxy for more disadvantaged environments). |
| Newman & Holupka, 2016 [68] (Peer-reviewed article) | Investigate whether assisted housing (Housing Choice Vouchers, public housing or funding to private promoters, such as Low-Income Housing Tax Credit) has effects on child welfare, focusing on cognitive, behavioral and health outcomes in adolescents. | **Country:** USA  **CAA age:** 3-17 years  **Level of intervention:** Structural, Intermediate | **Public housing:** Government-owned buildings in which all units are subsidized, tenants typically pay 30% of their monthly income in rent, and eligibility and allocation are determined by local housing authorities based on waiting lists and priority criteria.  **Housing vouchers:** The Housing Choice Voucher (HCV) program provides direct rental subsidies to families, allowing them to choose and rent housing in the private market. Families contribute with approximately 30% of their monthly income.  **Subsidies to private developers of affordable housing:** Low-Income Housing Tax Credit (LIHTC); the program incentivizes developers to build rental housing for low-income households by offering tax credits. Tenants typically pay 30% of either 50% or 60% of the Area Median Income (AMI), meaning rent is based on set income thresholds rather than the tenant’s actual income. However, Housing Choice Vouchers can be used to help cover rent in LIHTC units. These developments are often concentrated in high-poverty neighborhoods. | **Quantitative** (longitudinal with comparison group)  **N:** IG n=194 (receiving any kind of housing assistance); CG n=215 (no housing assistance)  **Number of interventions studied:** 3  **Period covered by analysis:** 1995-2007 | **Survey:** American Community Survey (ACS), objective data on neighborhood; health assessment of participating CAA  **Validated questionnaires:** Revised Tests of Achievement (W-J), for reading comprehension and applied mathematics; Behaviour Problems Index (BPI)  **Administrative records:** HUD’s assisted housing data;  Data on physical inspections of public housing (made by the HUD)  **Population census:** Data on neighborhood characteristics | 80% | **PHYISICAL HEALTH**  **Health conditions:** Living in assisted housing during childhood had no significant effects on the health outcomes of adolescents.  **MENTAL HEALTH**  **Behavior:** Living in assisted housing during childhood had no significant effects on the behavioral outcomes of adolescents. However, the quantile regression analyses showed that living in assisted housing had negative effects on adolescents at the lower end of the outcome distributions: children with better behavior experienced a reduction in behavioral problems, while those with worse behavior experienced an increase in these problems.  **EDUCATIONAL OUTCOMES**  **Short-term academic metrics:** No significant results, but the effects were positive on cognition of children and adolescents with high performance and negative for those with low performance when quantile analysis was performed. |
| Pergamit et al., 2017 [71] (Peer-reviewed article) | To evaluate the impact of the Family Unification Program (FUP) on the results of the child welfare system. Specifically, it is examined whether granting housing vouchers to families with children at risk of entering the shelter or who are already separated from their parents can reduce the need to separate children from their homes; accelerate family reunification in cases of reception; reduce the time of participation in the protection system, and reduce the number of new reports of abuse or neglect. | **Country:** USA  **CAA age:** Unspecified  **Level of intervention:** Intermediate | **Housing vouchers:** Family Unification Program (FUP); it provides housing vouchers to families with children at risk of entering or remaining in foster care due to inadequate housing. It supports (1) families facing imminent foster care placement, (2) families delaying reunification due to housing issues, and (3) young adults (18-21) transitioning from foster care without stable housing. Families can rent from private landlords, contributing up to 30% of their income, with 60-120 days to secure suitable housing. | **Quantitative** (longitudinal with comparison group)  **N:** IG n=570 (receiving housing vouchers); CG n=258 (no housing assistance)  **Number of interventions studied:** 1  **Period covered by analysis:** 2010-2012 | **Administrative records:** Data from the child and adolescent protection system; Referral forms to FUP (demographic information, housing situation, etc.) | 60% | **MALTREATMENT OUTCOMES:** Among families in preservation (those aiming to avoid separation from their children), there was an 18.5% reduction in substantiated abuse reports in Portland, and a 19.3% reduction in any type of new report (substantiated and unsubstantiated) in San Diego.  Among families in reunification, there was a 57% increase in unsubstantiated reports and a 24.8% increase in substantiated reports in Portland. In contrast, San Diego saw a 15.5% reduction in new reports. |
| Ports et al., 2018 [48] (Peer-reviewed article) | To examine the impact of the Low-Income Housing Tax Credit (LIHTC) program on rates of child abuse and neglect and unintentional injuries among children under four years old in Georgia, USA, from 2005 to 2015. | **Country:** USA  **CAA age:** 0-17 years  **Level of intervention:** Intermediate | **Subsidies to private developers of affordable housing:** Low-Income Housing Tax Credit (LIHTC); the program incentivizes developers to build rental housing for low-income households by offering tax credits. Tenants typically pay 30% of either 50% or 60% of the Area Median Income (AMI), meaning rent is based on set income thresholds rather than the tenant’s actual income. However, Housing Choice Vouchers can be used to help cover rent in LIHTC units. These developments are often concentrated in high-poverty neighborhoods. | **Quantitative** (longitudinal with comparison group)  **N:** 159 (Georgia counties); IG n=unspecified (counties with LIHTC units); CG n=unspecified (counties with few or any LIHTC units)  **Number of interventions studied:** 1  **Period covered by analysis:** 2005-2015 | **Administrative records:** HUD’s data on LIHTC users; National Child Abuse and Neglect Data System (NCANDS)  **Public health records:** OASIS System of Georgia (urgency care visits)  **Population census:** Data on socio-economic level, ethnic composition, etc. | 80% | **MALTREATMENT OUTCOMES:** In the bidirectional fixed effects model, which controlled for both county and temporal effects, no significant associations were found between the LIHTC program and any of the analyzed outcomes (child abuse, neglect, or unintentional injuries). |
| Rosero, 2012 [69] (Doctoral thesis) | Analyze how a housing aid program ("ABC") influence school enrollment, child labor, and poverty reduction, with a focus on the underlying mechanisms that explain these effects. | **Country:** Ecuador  **CAA age:** Unspecified  **Level of intervention:** Intermediate | **Housing vouchers:** The ABC program of Ecuador is aimed at poor families, providing grants in the form of housing vouchers of 5,000 USD for the purchase of a new home or the improvement of an existing one. Families must contribute 10% of the home's value in personal savings, with the remainder financed through a mortgage loan (private sector). The families are within the 60% poorest of the country. The program has a hybrid model (subsidy + savings + credit) that promotes access to homeownership rather than renting. | **Quantitative** (longitudinal with comparison group)  **N:** 3.304; IG n=unspecified (receiving a housing voucher); CG n=unspecified (no housing assistance)  **Number of interventions studied:** 1  **Period covered by analysis:** 2007-2009 | **Survey:** SELBEN panel (socio-economic data on households before and after intervention, with access to basic services, education, child labour...)  **Administrative records:** Data from the Ministry of Housing (history of applications and concessions) | 80% | **EDUCATIONAL OUTCOMES**  **Long-term educational attainment:** A significant increase of 4.6% in school enrollment for CAA aged 15-18 (post-compulsory age), reducing school dropout by 19%. No effect on younger children (6-14 years).  A 4.4% decrease in child labor for children and adolescents aged 15-18, equivalent to a 21% lower likelihood of working. |
| Sandel et al., 2016 [63] (Report) | Examine whether affordable and stable housing (through rental assistance) improves the health of children in vulnerable families, especially those with food insecurity, and how these investments can reduce the health costs associated with housing instability. | **Country:** USA  **CAA age:** 0-1 years  **Level of intervention:** Structural, Intermediate | **Public housing:** Government-owned buildings in which all units are subsidized, tenants typically pay 30% of their monthly income in rent, and eligibility and allocation are determined by local housing authorities based on waiting lists and priority criteria.  **Housing vouchers:** The Housing Choice Voucher (HCV) program provides direct rental subsidies to families, allowing them to choose and rent housing in the private market. Families contribute with approximately 30% of their monthly income.  **Subsidies to private developers of affordable housing:** Low-Income Housing Tax Credit (LIHTC); the program incentivizes developers to build rental housing for low-income households by offering tax credits. Tenants typically pay 30% of either 50% or 60% of the Area Median Income (AMI), meaning rent is based on set income thresholds rather than the tenant’s actual income. However, Housing Choice Vouchers can be used to help cover rent in LIHTC units. These developments are often concentrated in high-poverty neighborhoods. | **Quantitative** (cross-sectional with comparison group)  **N:** IG n=~500 (receiving housing assistance); CG n=~1.500 (no housing assistance)  **Number of interventions studied:** 3  **Period covered by analysis:** 2009-2014 | **Survey:** Survey for child caregivers  **Medical records:** Children’s health outcomes | 100% | **HEALTHCARE ACCES AND UTILIZATION**  **Hospitalizations:** 43% reduction in the likelihood of child hospitalization for families that received rental assistance during the prenatal period. Estimated savings of 20 million dollars in healthcare costs due to 1,200 hospitalizations avoided in 2015 because of rental assistance. |
| Schwartz et al., 2019 [64] (Peer-reviewed article) | Estimate the short-term impact of Housing Choice Vouchers on school performance of students from public schools in New York City, comparing their performance before and after receiving the intervention. | **Country:** USA  **CAA age:** 8-14 years  **Level of intervention:** Intermediate | **Housing vouchers:** The Housing Choice Voucher (HCV) program provides direct rental subsidies to families, allowing them to choose and rent housing in the private market. Families contribute with approximately 30% of their monthly income. | **Quantitative** (longitudinal without comparison group)  **N:** 32.671  **Number of interventions studied:** 1  **Period covered by analysis:** 2007-2009 | **Survey:** American Community Survey (ACS), for neighborhood characteristics  **Administrative records:** HUD’s housing voucher user data from 2002-2012; NYC Department of Education; NYC Department of Finance; NYC Department of Buildings | 100% | **EDUCATIONAL OUTCOMES**  **Short-term academic metrics:** Students in the HCV program have grades with a standard deviation of 0.048-0.058 better in English Language Arts (ELA) and Mathematics in the years following the receipt of the housing voucher. The positive impact is present among students who move after receiving the voucher, but also among those who do not.  Positive effects are concentrated among Asian, Hispanic, and White students, while the impact is nearly null among African American students. There are no differences between boys and girls. |
| Turcotte et al., 2018 [72] (Peer-reviewed article) | Evaluate whether government-assisted housing versus market-rate housing influences the frequency of asthma symptoms or quality of life scores among low-income urban children with asthma. | **Country:** USA  **CAA age:** 0-15 years  **Level of intervention:** Structural | **Public housing:** Government-owned buildings in which all units are subsidized, tenants typically pay 30% of their monthly income in rent, and eligibility and allocation are determined by local housing authorities based on waiting lists and priority criteria. | **Quantitative** (longitudinal with comparison group)  **N:** IG n=121 (living in public housing); CG n=136 (living in market-rate housing)  **Number of interventions studied:** 1  **Period covered by analysis:** 2009-2012 | **Survey:** Children’s Health Survey for Asthma (CSHA) | 60% | **PHYSICAL HEALTH**  **Health conditions:** Children in public housing had better asthma scores at baseline, with a 76.8/100 score in physical health, which had a score of 67.6.  **MENTAL HEALTH**  **Psychological distress:** Children in public housing had better family emotional health scores, with 74.8/100, compared to the group without assistance, which had a score of 71.6.  **HEALTHCARE ACCES AND UTILIZATION**  **Hospitalizations:** Children in public housing had fewer hospitalizations compared to the CG. |
